# Depression in left-behind children: a network analysis and chained mediation of attachment and emotion regulation

**DOI:** 10.1186/s40359-026-04848-0

**Published:** 2026-05-28

**Authors:** Kemeng Li, Ting Xu, Lewen Shang, Yi Chen, Wenjie Dou, Baoming Li, Zhong Yang

**Affiliations:** 1 Laboratory of Infant Development and Childcare, Hangzhou Normal University, Hangzhou, China; 2 Institute of Brain Science and Department of Physiology, School of Basic Medical Sciences, Hangzhou Normal University, 2318 Yuhangtang Road, Yuhang District, Hangzhou, Zhejiang China; 3 Department of Psychology, Jing Hengyi School of Education, Hangzhou Normal University, Hangzhou, China

**Keywords:** Left-behind children, Attachment, Emotion regulation, Depression, Network analysis

## Abstract

**Background:**

Urbanization has swelled the ranks of left-behind children (LBC), whose mental health — especially depression — now draws mounting global concern. Previous studies have shown that LBC are more prone to develop insecure parental attachments and tend to adopt maladaptive emotion regulation strategies, both established predictors of depression.

**Objective:**

Investigate the specific pathways through which attachment and emotion regulation mediate the impact of left-behind experiences on depression.

**Participants and setting:**

In three middle schools in Qiqihar, a total of 1,125 children aged 12–17 were sampled, including 573 LBC and 552 non‑left‑behind children (NLBC).

**Methods:**

The questionnaire survey includes a Left-behind Status Questionnaire, the Inventory of Parent and Peer Attachment (IPPA), the Emotion Regulation Questionnaire for Children and Adolescents (ERQ-CA), and the Children’s Depression Inventory (CDI).

**Results:**

(1) LBC scored significantly lower than NLBC in maternal attachment, paternal attachment, and cognitive reappraisal, while scoring significantly higher in expressive suppression and depression; (2) LBC showed network structure distinct from NLBC, with maternal attachment showing lower centrality and weaker coupling with paternal attachment; (3) LBC with both parents absent showed network structure and global strength distinct from NLBC, along with weaker maternal-paternal attachment correlation; (4) all four mediating pathways exhibited significant chain effects.

**Conclusions:**

(1) Left-behind experiences are associated with weakened parent-child attachment, reduced cognitive reappraisal, increased expressive suppression, and a heightened risk of depression; (2) In LBC, maternal attachment shows lower centrality, and the coupling between maternal and paternal attachment is significantly weaker, indicating a less coordinated parental attachment system. (3) The absence of both parents has a far greater effect on reshaping children’s psychological networks than the absence of the father alone. (4) parent-child attachment and emotion regulation mediated the association between left-behind experiences and depression.

**Supplementary Information:**

The online version contains supplementary material available at 10.1186/s40359-026-04848-0.

## Introduction

In China, left-behind children (LBC) are officially defined as children under 18 years old who remain at their original residence after one or both parents migrate for work, resulting in separation for at least 6 months [1]. Globally, this phenomenon affects hundreds of millions of children [2], with approximately 61 million in China [3]. Compared to non-left-behind children (NLBC), LBC exhibit more mental health problems due to impaired parent-child relationships, diminished parental support, and deficient parental guidance [4]. Among the most prevalent and clinically significant mental health challenges faced by LBC is depression [5].

Depression is characterized by persistent feelings of sadness, anxiety, hopelessness, and often includes physical symptoms, such as insomnia [6]. The prevalence of depression in LBC was significantly higher than in NLBC [7]. This disparity may be attributed to prolonged parent-child separation, which undermines the development of secure attachment and increases vulnerability to negative emotions [8, 9]. In addition, due to parental absence, LBC demonstrate reduced adoption of cognitive reappraisal strategies and more frequent use of expressive suppression, consequently exacerbating depressive symptoms [10].

Attachment plays a crucial role in the development of depression among children. Attachment is the emotional bond between child and caregiver (typically the parents), through which the child seeks closeness, protection and safety under stress and danger [1]. Secure parent-child attachment provides a “safe base” that enables children to receive emotional support and buffers stress [11]. In contrast, insecure parent-child attachment can lead children to develop negative self-representations (e.g., “I am unlovable”) and other-representations (e.g., “others are unreliable”), fostering helplessness and self-blame, thereby increasing vulnerability to depression [12, 13]. Ecological systems theory further posits that the microsystems like peer groups have a direct impact on children’s development [14], highlighting the growing importance of peer attachment with age [15]. Peer attachment is an emotional connection formed through sustained peer interaction, providing adolescents intimacy, warmth, support, and a buffer against negative family influences [16]. Therefore, peer attachment may serve as an alternative protective factor in alleviating depression among LBC.

Emotion regulation also plays a significant role in the formation and development of depression among LBC. Emotion regulation refers to the process by which individuals consciously influence the generation, experience, and expression of emotions, primarily through cognitive reappraisal and expressive suppression [17]. Cognitive reappraisal is an adaptive strategy that adjusts cognitions of emotionally triggering situations to alter emotional experiences [18], whereas expressive suppression is a maladaptive strategy involving the inhibition of emotional expression to regulate subjective experiences [19]. NLBC are more inclined to use cognitive reappraisal: stable family environments with steady parental support foster positive framing and blunt negative emotions [20]. In contrast, due to their familial circumstances, LBC may provoke conflict or remain misunderstood when expressing their emotions, leading them to rely more on expressive suppression, thereby increasing the risk of depression [21].

Previous studies have found that attachment styles are closely associated with the selection of emotion regulation strategies [22]. Individuals with secure attachments tend to employ adaptive strategies—such as cognitive reappraisal, problem-solving, and seeking support, which facilitate effective stress management and mitigation of negative emotions [23, 24]. In contrast, those with insecure attachment patterns are more likely to rely on maladaptive strategies when faced with stress, such as concealing their emotions and avoiding the expression of negative feelings [24]. Although such strategies may help avoid emotional conflicts in the short term, they can lead to emotional accumulation and an increased risk of depression in the long term [25]. Therefore, based on previous findings that left-behind experiences may contribute to insecure attachment and maladaptive emotion regulation, we speculated that attachment and emotion regulation may serve as chained mediators in the association between left-behind experience and depression.

In this study, attachment was categorized as maternal attachment, paternal attachment, and peer attachment, while emotion regulation includes cognitive reappraisal and expressive suppression. Previous studies have primarily relied on traditional methods such as correlation or regression analyses, which often assume unidirectional or latent relationships between variables, potentially oversimplifying the complex interplay among psychological constructs [26]. To address this limitation, this study primarily employed two complementary methods: network analysis and chain mediation analysis. Network analysis serves primarily an exploratory function. It enables visualization of the complex network among attachment, emotion regulation, and depression, facilitates the identification of key nodes that may drive network dynamics [27], and allows comparison of overall network structures as well as specific edge weights between left-behind children and non-left-behind children [28]. In contrast, chain mediation analysis primarily serves as a confirmatory tool. It validates whether the key nodes identified through network analysis function as hypothesized mediators, and examines specific theoretically derived directional pathways [27, 29]. Therefore, combining these two methods allows for a more detailed and structured understanding of the potential pathways through which attachment and emotion regulation influence depression in left-behind children.

**Fig. 1:**
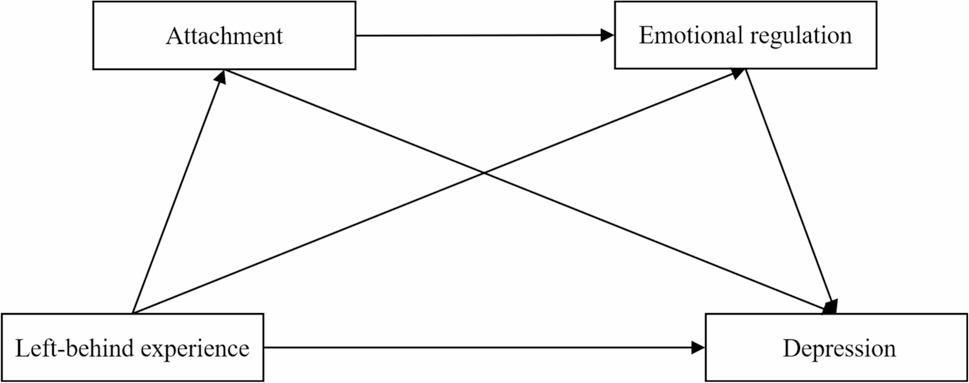
Chain-mediated model diagram

Overall, this study employed a questionnaire survey method to collect data on basic demographic information, maternal attachment, paternal attachment, peer attachment, cognitive reappraisal, expressive suppression, and depression scores among LBC and NLBC. Statistical analyses included multivariate analysis of variance, network analysis, and chain mediation models. We hypothesize that: (1) left-behind experience would predict lower attachment security and a greater tendency to adopt maladaptive emotion regulation strategies; (2) the network structures linking maternal, paternal, and peer attachment with emotion regulation strategies and depressive symptoms will demonstrate significant between-group differences (LBC vs. NLBC, LBC with both parents absent vs. NLBC); (3) attachment and emotion regulation would serve as chain mediators between left-behind experience and depression (see Fig. 1).

## Methods

### Participants

The participants were recruited from three middle schools in Qiqihar City, Heilongjiang Province, China. We used a cluster sampling method to collect 3,953 samples. First, we extracted 1,069 questionnaires from LBC and 2,884 questionnaires from NLBC. Then, we removed invalid questionnaires from the 1,069 LBC questionnaires, leaving 609 valid responses. Since the impact of being left-behind is more pronounced in early childhood, we excluded 36 samples of LBC whose parents migrated after the age of 12 to ensure that the participants were more representative. Finally, 573 valid samples of LBC (271 boys, M_age_ = 14.25 years, SE = 1.34, range from 12 to 17 years) were included, and samples of 552 NLBC (257 boys, M_age_ = 14.16 years, SE = 1.34, range from 12 to 17 years) were matched using propensity score matching based on the demographic characteristics of the LBC. The flowchart of the sample screening process is shown in Supplementary Figure S1. Chi-square test results showed no significant differences in demographic information between the two groups (Supplementary Table S1). They voluntarily participated and signed an informed consent form before completing the questionnaire. Additionally, the study was approved by the Research Ethics Committee of Hangzhou Normal University.

### Materials

#### Left-behind Status Questionnaire

We used a self-developed Left-behind Status Questionnaire to collect detailed information about LBC, such as the initial time and duration of parents working away from home, the situation of those left behind to provide care, and the frequency and methods of communication with their parents (see Supplementary Material). 

#### Inventory of Parent and Peer Attachment, IPPA

The Inventory of Parent and Peer Attachment (IPPA; see Supplementary Material), was developed by Armsden et al. in 1987 and revised in 1991 [30]. The revised IPPA was designed for adolescents aged 12 to 20 years, and was used to measure of participants’ maternal attachment, paternal attachment, and peer attachment, with the results yielding continuous attachment quality scores. Each subscale consists of 25 items rated on a 5-point Likert scale. The theoretical score range for each attachment is 25 to 125, with higher scores indicating greater attachment quality. Each subscale is further divided into three dimensions: trust, communication, and alienation. The trust dimension measures the degree of mutual understanding, respect, and trust; the communication dimension measures the degree and quality of verbal communication with attachment partners; and the alienation dimension measures the degree of anger towards the attachment partner and emotional separation and isolation from the attachment partner. The Cronbach’s α coefficients of IPPA revealed acceptable internal consistency (α_Maternal Attachment_ = 0.932, α_Paternal Attachment_ = 0.937, α_Peer Attachment_ = 0.923).

#### The Emotion Regulation Questionnaire for Children and Adolescents, ERQ-CA

The Emotion Regulation Questionnaire for Children and Adolescents (ERQ-CA; see Supplementary Material), was developed by Eleonora Gullone and John Taffe in 2012 and revised based on adult measurements [31]. The ERQ-CA was used to measure individual’s habitual use of two common emotion regulation strategies: cognitive reappraisal (CR) and expressive suppression (ES). It consists of 10 items, with six assessing CR and four assessing ES. The questionnaire is scored on a 5-point Likert scale, with higher scores indicating greater use of the corresponding strategy. The Cronbach’s α coefficients of the ERQ-CA revealed acceptable internal consistency (α_CR_ = 0.854, α_ES_ = 0.707).

#### Children’s Depression Inventory, CDI

The Children’s Depression Inventory (CDI) was developed by Wu et al. in 2010 (see Supplementary Material), and was designed for adolescents aged 12 to 20 years [32]. It is the Chinese translated version of the Kovacs Children’s Depression Scale. The scale consists of 27 items and is divided into 5 subscales: anhedonia, negative mood, negative self-esteem, ineffectiveness, and interpersonal problem. Each item offers three options scored from 0 to 2, where 0 indicates the least severe depressive symptoms, 1 indicated moderate symptoms, and 2 indicates the most severe symptoms, yielding a total score of 54 points, with higher scores indicating more severe depression [33]. The CDI scale includes 13 reverse-scored items and some overlapping items, which are deliberately designed for children and adolescents to test the attitude of answering questions and the authenticity of answering questionnaires. The Cronbach’s alpha coefficient of the CDI was 0.885.

### Procedures

This research was conducted by teachers who had been systematically trained to ensure they could accurately guide the students in completing the questionnaire. Prior to administering the questionnaire, the teachers first explained the purpose and usage of the questionnaire to the students to guarantee their understanding. The questionnaire was then administered and collected within the class.

After collection, the data underwent a thorough screening and cleaning process to ensure its completeness and accuracy. Subsequently, the valid data were input into a database. Reverse-scored items (Items 2, 5, 7, 8, 10, 11, 13, 15, 16, 18, 21, 24, and 25 in the CDI; Items 3, 6, 8, 9, 10, 11, 14, 17, 18, and 23 in parent attachment; and Items 4, 5, 9, 10, 11, 18, 22, and 23 in peer attachment) were recoded prior to analysis to ensure a consistent scoring direction across all items. Any incomplete, logically inconsistent, or obviously anomalous questionnaire records were discarded.

### Data analyses

First, to explore the impact of left-behind experiences (NLBC vs. LBC) on attachment, emotion regulation, and depression levels, we conducted multivariate analysis of variance (MANOVA). Analyses were performed using SPSS 26.0 with a statistical significance of α = 0.05 (two-tailed). All the results were corrected using Bonferroni tests if there were more than three groups in the post hoc analyses and the simple effect analyses.

Second, to explore the bidirectional relationship among attachment, emotion regulation, and depression levels, we conducted regularized partial correlation network analysis using the “qgraph” [34] and “bootnet” packages [35] in the R platform. We constructed a regularized partial correlation network based on data pertaining to the attachment, emotion regulation, and depression of left-behind children. Subsequently, we calculated centrality measures (strength, closeness, betweenness, and expected influence) for the left-behind children’s network, visually demonstrating the strength of associations among variables and their relative importance within the network. Furthermore, we compared the structural differences between distinct groups (NLBC group, father absent group, and father & mother absent group) and conducted a network comparison test (NCT) to further analyze the relationship patterns among these groups. The steps of network analysis were presented in Supplementary Material.

Finally, to validate the chain mediation effects of attachment and emotion regulation on the experiences of left-behind children affecting depression, we employed PROCESS macro Model 6 for mediation analysis. The models tested sequential pathways where either maternal attachment (MA), paternal attachment (PA), or peer attachment (PeA) as the first mediator, followed by either cognitive reappraisal (CR) or expressive suppression (ES) as the second mediator. The analysis utilized ordinary least squares to estimate model parameters, generating unstandardized coefficients to evaluate direct and indirect effects, with statistical significance at α = 0.05 (two-tailed). Furthermore, a bootstrapping approach with 5,000 resamples was implemented to establish 95% confidence intervals, where direct and indirect effects were identified when the confidence intervals did not include zero.

## Results

### The impact of the left-behind experience on attachment, emotion regulation, and depression

Multiple analysis of variance (MANOVA) showed that the combined effect of left-behind experience on attachment, emotion regulation and depression was significant, Pillai’s Trace = 0.02, *F*(6, 1118) = 4.03, *p* < .001, *η*_*p*_ *²* = 0.02. Follow-up univariate ANOVA analysis showed that, the ANOVA results showed that non-left-behind children scored higher in maternal attachment, paternal attachment, and cognitive reappraisal, but lower in expressive suppression and depression compared to left-behind children. However, there was no significant difference in peer attachment between the two groups. Detailed statistics, including *F* and *p*-values, were presented in Table 1.

**Table 1:** A multivariate analysis of variance on the effects of left-behind experience on attachment, emotion regulation, and depression (Mean ± SD)

Dependent variables	NLBC (*n* = 552)	LBC (*n* = 573)	F (1, 1123)	*p*	η_*p*_ ²
Maternal attachment	95.44 ± 18.81	92.93 ± 17.74	5.30	0.022	0.005
Paternal attachment	91.38 ± 20.18	86.69 ± 19.36	15.78	< 0.001	0.014
Peer attachment	94.11 ± 16.04	92.80 ± 15.84	1.89	0.169	0.002
Cognitive reappraisal	20.87 ± 5.53	20.21 ± 5.24	4.23	0.040	0.004
Expressive suppression	12.43 ± 3.79	12.91 ± 3.44	4.88	0.027	0.004
Depression	12.55 ± 8.11	14.37 ± 8.11	14.12	< 0.001	0.012

*NLBC *non-left-behind children, *LBC *left-behind children

### The relationship among attachment, emotion regulation, and depression

#### The networks of NLBC and LBC groups

**Fig. 2:**
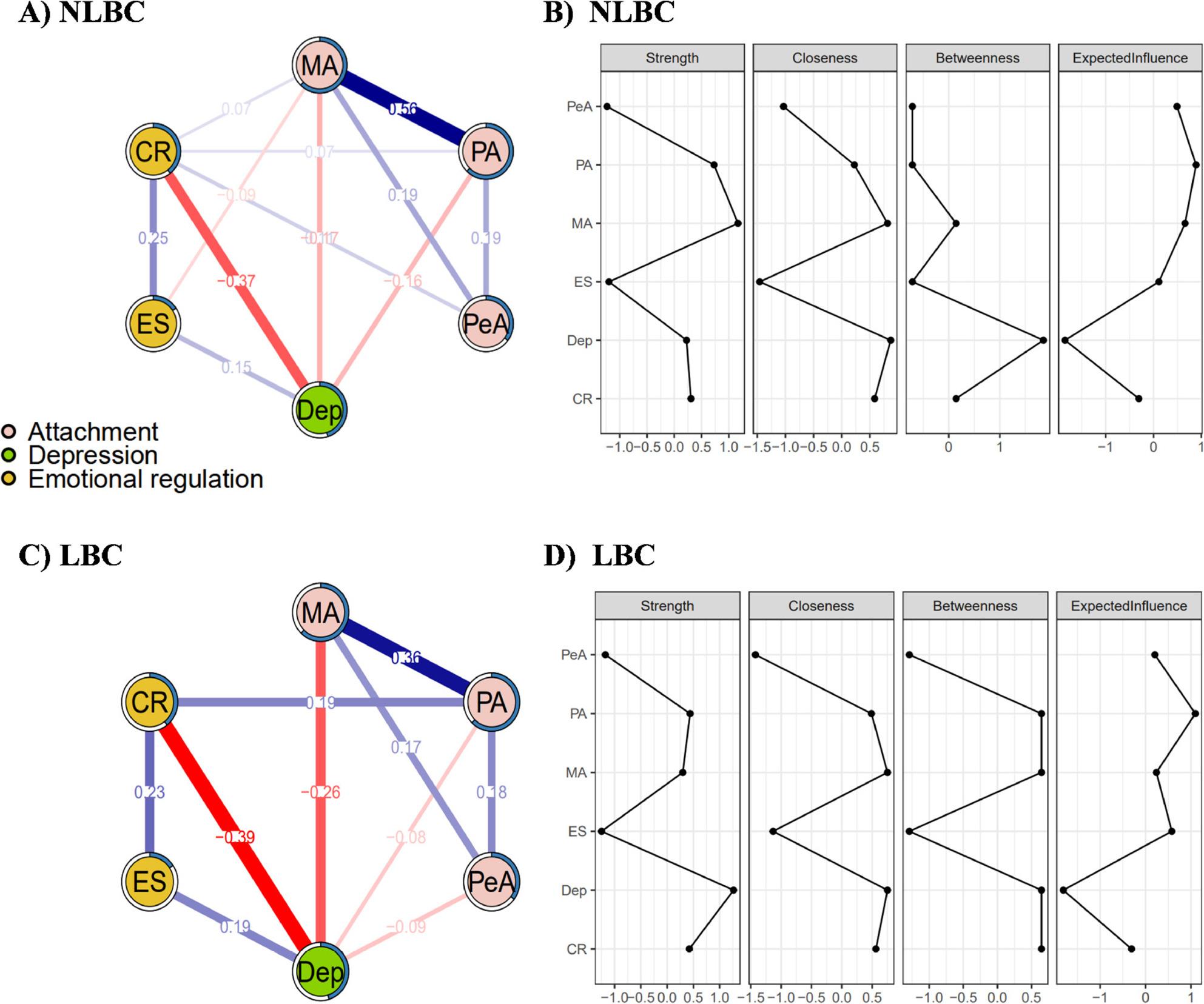
**A** The network and **B** its standardized center estimates of non-left-behind children (NLBC). **C** The network and **D** its standardized center estimates of left-behind children (LBC). MA, maternal attachment; PA, paternal attachment; PeA, peer attachment; CR, cognitive reappraisal; ES, expressive suppression; Dep, Depression

The network structures and centrality metrics for the NLBC and the LBC were presented in Fig. 2. Comparative analysis of the networks indicated that there were significant differences in network structures between the NLBC group and the LBC group (*T* = 0.21, *p* = .008), but no significant differences in global connectivity strength (*S* = 0.15, *p* = .228). In addition, there were significant differences in the MA-PA edges between the two groups (*E* = 0.21, *p* = .030). Finally, there was a significant difference in the strength centrality of MA between the two groups (*C* = − 0.26, *p* = .024). Other edge weights and centrality metrics showed no significant differences between the two groups. The centrality metrics showed that, in the NLBC network, MA exhibited the highest strength centrality, while PA exhibited the highest expected influence. In the LBC network, Dep exhibited the highest strength centrality, and PA exhibited the highest expected influence. Supplementary Figures S2 and S3 presented the stability and accuracy of the network for the LBC and NLBC. Supplementary Table S2 presented the CS coefficients of centrality indicators. Supplementary Tables S3 and S4 presented the node centrality of the LBC and NLBC. Supplementary Table S7 showed the differences in edge weights between the two subgroups.

#### The networks of NLBC, father absent, and father & mother absent groups

**Fig. 3:**
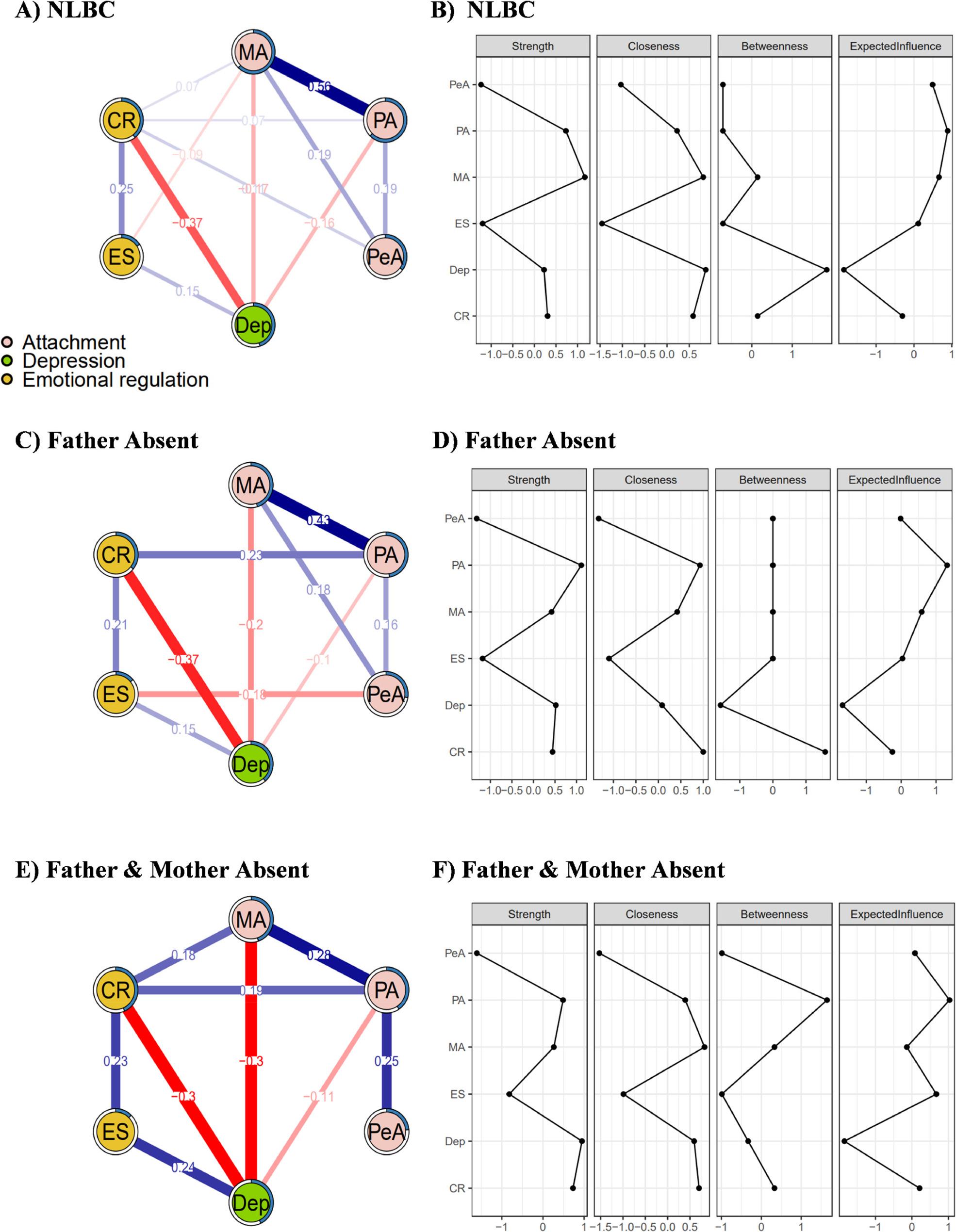
**A** The network and **B** its standardized center estimates of non-left-behind children (NLBC). **C** The network and **D** its standardized center estimates of father absent group. **E** The network and **F** its standardized center estimates of father & mother absent group. MA, maternal attachment; PA, paternal attachment; PeA, peer attachment; CR, cognitive reappraisal; ES, expressive suppression; Dep, Depression

We estimated the network structures of the NLBC group, the father absent group, and the father & mother absent group (Fig. 3). The comparative analysis of the networks indicated that there were significant differences in network structure between the father & mother absent group and the NLBC group (*M* = 0.30, *p* = .002), and the two groups also showed significant differences in global connectivity strength (*S* = 0.45, *p* = .026). Finally, there were significant differences between the two groups on the MA-PA edges (*E* = 0.30, *p* = .015). Notably, centrality metrics did not show significant differences in this comparison, and no other edge weights differed significantly between the two groups. Centrality indicators suggested that in the NLBC network, MA exhibited the highest strength centrality, while PA exhibited the highest expected influence. In the father absent group network, PA exhibited the highest expected influence. In the father and mother absent group network, Dep exhibited the highest strength centrality, and PA exhibited the highest expected influence. Supplementary Figures S4 and S5 presented the stability and accuracy of the network for the father absent group and father and mother absent group. Supplementary Tables S5 and S6 presented the node centrality of the father absent group and father and mother absent group. Supplementary Table S8 presented the differences in edge weights among the three groups.

### Chain mediation model testing

The means, standard deviations, and Pearson correlation matrix for all variables were presented in Table 2. The results indicated that left-behind experience was significantly negatively correlated with maternal attachment, paternal attachment, and cognitive reappraisal, while it showed significant positive correlations with expressive suppression and depression. Meanwhile, maternal attachment, paternal attachment, peer attachment, and cognitive reappraisal were significantly positively correlated with each other. Expressive suppression was positively associated with depression, while all three types of attachment showed significant negative correlations with both expressive suppression and depression. Furthermore, cognitive reappraisal was positively correlated with expressive suppression, but negatively correlated with depression.

**Table 2:** Descriptive statistics and correlations among various variables (*N* = 1125)

Variables	M ± SD	1	2	3	4	5	6
1 Left-behind experience	-						
2 Maternal attachment	94.16 ± 18.31	− 0.07^**^					
3 Paternal attachment	88.99 ± 19.90	− 0.12^**^	0.67^**^				
4 Peer attachment	93.45 ± 15.94	− 0.04	0.47^**^	0.48^**^			
5 Cognitive reappraisal	20.53 ± 5.39	− 0.06^*^	0.40^**^	0.43^**^	0.32^**^		
6 Expressive suppression	12.68 ± 3.62	0.07^*^	− 0.14^**^	− 0.15^**^	− 0.14^**^	0.15^**^	
7 Depression	13.48 ± 8.16	0.11^**^	− 0.53^**^	− 0.49^**^	− 0.36^**^	− 0.52^**^	0.18^**^

Left-behind experience coding: 0 = non-left-behind children, 1 = left-behind children

*M ± SD *Means ± Standard Deviation

^*^p < .05; ^**^p < .01; ^***^p < .001

Since no significant correlation was found between peer attachment and left-behind experience (LBE), we included only maternal attachment (MA) or paternal attachment (PA) as the first mediator, followed by either cognitive reappraisal (CR) or expressive suppression (ES) as the second mediator. LBE was set as the independent variable, and depression (Dep) as the dependent variable, resulting in four mediation model combinations: LBE→MA→CR→Dep, LBE→PA→CR→Dep, LBE→MA→ES→Dep, and LBE→PA→ES→Dep. Table 3 presents the results of the specific and total indirect effect and the total effect of maternal attachment, paternal attachment on Depression through cognitive reappraisal and expressive suppression. The results of all path coefficients can be found in Supplementary Table S9 and S10.

**Table 3:** Specific and total indirect effect and the total effect of maternal attachment, paternal attachment on depression through cognitive reappraisal and expressive suppression

Cognitive Reappraisal			Expressive Suppression		
Paths	β (SE)	CI	Path	β (SE)	CI
MA→CR			MA→ES		
Total effect	1.82^***^ (0.48)	[0.87, 2.77]	Total effect	1.82^***^ (0.48)	[0.87, 2.77]
Total direct effect	1.03^**^ (0.38)	[0.29, 1.78]	Total direct effect	1.14^**^ (0.41)	[0.33, 1.95]
Total indirect effect	0.78 (0.31)	[0.18, 1.37]	Total indirect effect	0.68 (0.26)	[0.18, 1.2]
LBE→MA→Dep	0.42 (0.19)	[0.06, 0.79]	LBE→MA→Dep	0.57 (0.25)	[0.1, 1.08]
LBE→CR→Dep	0.20 (0.16)	[-0.12, 0.54]	LBE→ES→Dep	0.10 (0.06)	[-0.004, 0.22]
LBE→MA→CR→Dep	0.16 (0.07)	[0.02, 0.31]	LBE→MA→ES→Dep	0.02 (0.01)	[0.002, 0.04]
PA→CR			PA→ES		
Total effect	1.82^***^ (0.48)	[0.87, 2.77]	Total effect	1.82^***^ (0.48)	[0.87, 2.77]
Total direct effect	0.81^*^ (0.39)	[0.05, 1.58]	Total direct effect	0.80 (0.42)	[-0.03, 1.63]
Total indirect effect	1 (0.29)	[0.43, 1.59]	Total indirect effect	1.02 (0.24)	[0.56, 1.49]
LBE→PA→Dep	0.62 (0.17)	[0.31, 0.96]	LBE→PA→Dep	0.90 (0.23)	[0.47, 1.36]
LBE→CR→Dep	0.07 (0.17)	[-0.26, 0.41]	LBE→ES→Dep	0.08 (0.06)	[-0.01, 0.21]
LBE→PA→CR→Dep	0.31 (0.09)	[0.14, 0.49]	LBE→PA→ES→Dep	0.03 (0.01)	[0.01, 0.06]

The total effects are identical across all models (*B* = 1.82) because all four chain mediation models share the same independent variable (LBE) and dependent variable (Dep); thus, the same total effect model (LBE → Dep without mediators) was estimated for each model

*LBE *left-behind experience, *MA *maternal attachment, *PA *paternal attachment, *CR *cognitive reappraisal, *ES *expressive suppression, *Dep *Depression, *SE *standard error, *CI *95% confidence interval

^*^p < .05; ^**^p < .01; ^***^p < .001

**Fig. 4:**
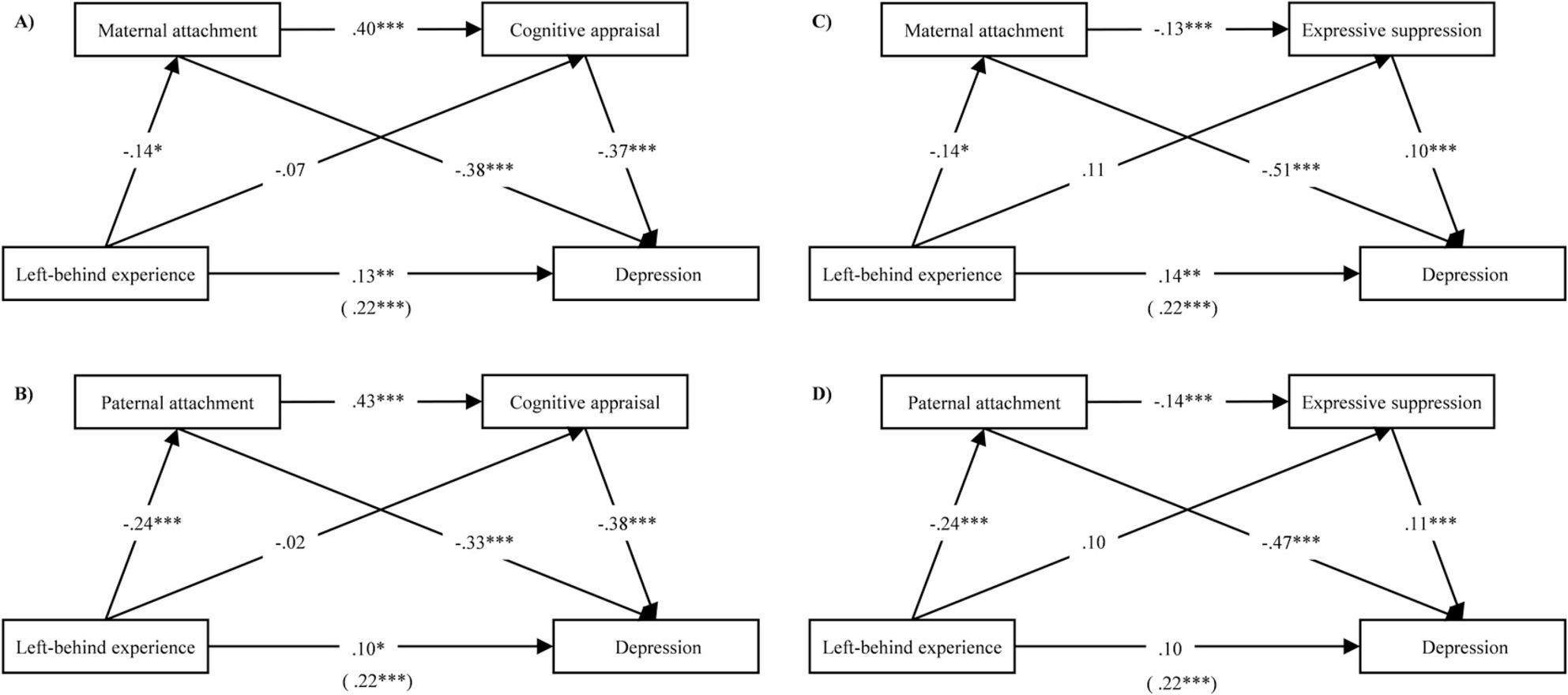
The chain mediation effects of **A** MA→CR, **B** PA→CR, **C** MA→ES and **D** PA→ES. MA, maternal attachment; PA, paternal attachment; CR, cognitive reappraisal; ES, expressive suppression. *** Significant at the .001 level. ** Significant at the .01 level. * Significant at the .05 level

For the chain mediation model with maternal attachment and cognitive reappraisal serve as mediating variables (see Fig. 4A), the total effect analysis showed that left-behind experience positively predicted depression (*B* = 1.82, *p* < .001). The direct effect remained significant even after accounting for the mediating effects of maternal attachment and cognitive reappraisal (*B* = 1.03, *p* < .001). The indirect pathway of the “LBE→MA→Dep” yielded a significant indirect effect (*B* = 0.42, *95% CI* [0.06, 0.79]). However, the confidence interval for the indirect pathway of the “LBE→CR→Dep” included zero (*B* = 0.20, *95% CI* [-0.12, 0.54]). Furthermore, the sequential mediation pathway of the “LBE→MA→CR→Dep” exhibited a significant chain effect, although its effect size was small (*B* = 0.16, *95% CI* [0.02, 0.31]).

For the chain mediation model with paternal attachment and cognitive reappraisal serve as mediating variables (see Fig. 4B), the total effect analysis showed that left-behind experience positively predicted depression (*B* = 1.82, *p* < .001). The direct effect remained significant even after accounting for the mediating effects of paternal attachment and cognitive reappraisal (*B* = 0.81, *p* = .038). The indirect pathway of the “LBE→PA→Dep” yielded a significant indirect effect (*B* = 0.62, *95% CI* [0.31, 0.96]). However, the confidence interval for the indirect pathway of the “LBE →CR→Dep” included zero (*B* = 0.07, *95% CI* [-0.26, 0.41]). Furthermore, the sequential mediation pathway of the “LBE→PA→CR→Dep” exhibited a significant chain effect (*B* = 0.31, *95% CI* [0.14, 0.49]).

For the chain mediation model with maternal attachment and expressive suppression serve as mediating variables (see Fig. 4C), the total effect analysis showed that left-behind experience positively predicted depression (*B* = 1.82, *p* < .001). The direct effect remained significant even after accounting for the mediating effects of maternal attachment and expressive suppression (*B* = 1.14, *p* = .006). The indirect pathway of the “LBE→MA→Dep” yielded a significant indirect effect (*B* = 0.57, *95% CI* [0.10, 1.08]). However, the confidence interval for the indirect pathway of the “LBE→ES→Dep” included zero (*B* = 0.10, *95% CI* [-0.004, 0.22]). Finally, the sequential mediation pathway of the “LBE→MA→ES→Dep” exhibited a significant chain effect, although its effect size was small (*B* = 0.02, *95% CI* [0.002, 0.04]).

For the chain mediation model with paternal attachment and expressive suppression serve as mediating variables (see Fig. 4D), the total effect analysis showed that left-behind experience positively predicted depression (*B* = 1.82, *p* < .001). The indirect pathway of the “LBE→PA→Dep” yielded a significant indirect effect (*B* = 0.90, *95% CI* [0.47, 1.36]). However, the confidence interval for the indirect pathway of the “LBE→ES→Dep” included zero (*B* = 0.08, *95% CI* [-0.01, 0.21]). Finally, the sequential mediation pathway of the “LBE→PA→ES→Dep” exhibited a significant chain effect, although its effect size was small (*B* = 0.03, *95% CI* [0.01, 0.06]).

## Discussion

This section was divided into four parts. First, the study found that left-behind children (LBC) exhibited lower quality of parent-child attachment, greater use of maladaptive emotion regulation strategies, and higher levels of depression compared to non-left-behind children (NLBC). Second, the study explored the relationships among attachment, emotion regulation, and depression, highlighting the central role of maternal attachment within the family system. Third, the study revealed that attachment and emotion regulation play a chain mediating role in the relationship between left-behind experience and depression. Finally, this study addressed some limitations and provided suggestions and directions for future research.

### The psychological impact of the left behind experience

Consistent with previous studies, this study found that left-behind experiences had a significant negative impact on children’s psychological development [36]. First, LBC exhibit higher depression, which may stem from prolonged parental absence that makes heightens helplessness under stress and reduces effective emotional processing [6]. Second, the disruption of early bonding in LBC is often accompanied by insecure attachments with parents [37]. Notably, peer attachment does not differ from NLBC, suggesting that LBC are capable of forming healthy relationships with peers [38]. Finally, prolonged parental absence further reduces opportunities for learning adaptive emotion regulation [21]. The absence of parents reduces the likelihood of children from learning emotional regulation strategies through observing their parents’ behavior, making it more difficult for them to engage in positive regulation by reappraising stressful events. Instead, they are more likely to interpret negative events as persistent and global threats [10]. In some left-behind families, emotional expression is perceived as a “burden” [39], therefore, LBC are more likely to adopt negative emotion regulation strategies such as avoidance, expressive suppression, or excessive reliance on others. Although these strategies may temporarily alleviate distress, they may ultimately have a negative impact on the development of long-term emotional competence [40].

### The psychological signatures in the networks of NLBC and LBC

The results of the network analysis showed that the centrality of maternal attachment was weaker in the network of LBC. Centrality reflects the influence of variables within a network [41]. The decrease in maternal attachment centrality suggests a reduced connection with other psychological variables such as emotion regulation and depression, which indicates that the emotional buffering function of the mother may be diminished among LBC [12]. Further analysis revealed that LBC whose fathers are absent while mothers remain present exhibit similar characteristics to NLBC. However, when both parents are absent, there is a significant reduction in maternal attachment centrality, indicating that distancing from the mother may weaken emotional connection [42].

The connection strength (edge weight) of maternal and paternal attachment is significantly lower in the psychological network of LBC compared to NLBC. In well-functioning families, parental attachment develops through co-parenting, resulting in functional coupling (for example, the mother offers emotional comfort and the father provides cognitive guidance), which is accompanied by higher connection strength [43]. However, for LBC, the correlation between maternal and paternal attachment levels is weaker (as reflected by the lower edge weight between MA and PA in the network), indicating that in the absence of daily co‑parenting interactions, these maternal and paternal attachment develop more independently and with less coordination [44]. Nevertheless, this remains speculative and requires longitudinal research to determine whether this pattern represents an adaptive reorganization or a risk factor for psychopathology. Furthermore, the amplification effect of spatial separation suggests that when parents work in different locations (e.g., the mother in the rural area and the father in another province), children need to establish differentiated psychological representations, which reduces parental attachment bonds [45].

Paternal attachment shows the highest expected influence in both LBC and NLBC networks. This suggests that paternal attachment, through its extensive network connections, has a significant impact on overall network functioning, potentially reflecting the unique role of fathers in providing cognitive guidance and behavioral regulation, whose absence may lead to far-reaching cascading effects [46, 47]. However, from NLBC to LBC, the core of network strength centrality shifts from maternal attachment to depression, reflecting a structural transition from a protective network to a risk network. This shift indicates that the left-behind experience is not merely a change in a single variable, but a systematic alteration in the organization of overall network functioning. The left-behind experience may make depression a key driving factor in the psychological network by reducing core protective resources (maternal attachment) [48].

### The chain mediating role of attachment and emotion regulation

The study found that parent-child attachment (maternal and paternal attachment) and emotion regulation (cognitive reappraisal and expressive suppression) played a chain mediating role in the relationship between left-behind experience and depression. These results indicate that insecure maternal and paternal attachments jointly constitute the initial stage of increased depression risk, suggesting that depression is not merely triggered by the breakdown of a single parent-child relationship, but rather arises from the overall impairment of the parental attachment system. Maternal attachment fosters children’s basic sense of security and emotional stability through soothing and caregiving [49], whereas paternal attachment is primarily associated with encouraging exploration, competitiveness, and the development of self-regulation [50]. As paternal and maternal attachments are equally important in the development of children’s emotional regulation systems, it is essential to strengthen left-behind children’s emotional bonds with both parents in order to reduce their risk of depression [51].

Subsequently, a decline in parent-child attachment quality is associated with a decrease in the use of adaptive strategies (such as cognitive reappraisal), while it is simultaneously associated with an increased reliance on maladaptive strategies (such as expressive suppression). This may be related to the fact that left-behind children receive less effective emotional guidance and support from insecure parent-child attachments, which is negatively correlated with the development of emotion regulation abilities [52]. When LBC tend to cope with negative emotions through expressive suppression rather than cognitive reappraisal, these emotions may accumulate over time, which is associated with increased psychological stress and a higher risk of depression [53]. This highlights the need to provide systematic guidance and training-such as emotion regulation courses, psychological counseling, and group counseling-to help LBC gradually adopt cognitive reappraisal strategies that enable them to understand and reframe stressors from a positive perspective [54]. These interventions can assist them in recognizing and reducing their overreliance on expressive suppression, while also encouraging healthy emotional expression [55].

### Limitations and future prospects

This study employed a cross-sectional design and revealed the complex associations among attachment, emotion regulation, and depression in the population of LBC through network analysis and chain mediation models. Although the results indicated that insecure parent-child attachment was associated with higher levels of depression via emotion regulation strategies (i.e., reduced cognitive reappraisal and increased expressive suppression), the cross-sectional nature of the study limits causal inference and prevents determination of the directional effects among these variables. While the chain mediation models were theoretically grounded in Attachment Theory, the observed directional pathways reflect the statistical decomposition of variance among variables, rather than actual temporal processes or causal sequences [56]. It is worth noting that existing literature has also indicated the possibility of reverse effects. For instance, a study on adolescents indicated that higher levels of depression were associated with a subsequent decline in peer attachment, suggesting that depression may, in turn, undermine attachment relationships [57]. Future research could adopt a longitudinal design, combining cross-lagged panel models with temporal network analysis to further elucidate the causal relationships and potential pathways among variables.

Although the internal attrition rate of LBC in this study (approximately 43%) is within an acceptable range, the possibility of selection bias cannot be completely ruled out. For example, sensitive topics such as family separation may trigger avoidance responses from participants [58], resulting in systematic differences in emotional stability, conscientiousness, or levels of depression between LBC who completed the entire questionnaire and those who did not. Nevertheless, we have controlled for observable demographic variables in propensity score matching, and after matching, the two groups were well balanced in covariates. Therefore, future researchers are recommended to use shortened measurement instruments or conducting surveys in stages to reduce fatigue effects [59]. In addition, adopting self-administered modes (such as online surveys) and enhancing privacy protection measures can reduce social desirability bias and improve response rates to sensitive questions [58].

The participants in this study were adolescents aged 12–17 from three middle schools in Qiqihar, Heilongjiang Province. Although demographic characteristics were controlled through a matching design, the diversity of the sample remains limited. There may be systematic regional differences in the left-behind patterns and socioeconomic conditions: Heilongjiang Province, as a major agricultural province, is dominated by father-absent families [60]; in contrast, economically developed areas such as the Yangtze River Delta, and Pearl River Delta show more “semi-left-behind” patterns with both parents absent [61]; meanwhile, western provinces like Gansu and Guizhou face severe resource constraints [62]. These regional differences may produce heterogeneity in the attachment network structures and mechanisms underlying depression. Future research should incorporate multi-regional samples, including rural areas in the eastern and western regions as well as coastal industrial zones, to verify the generalizability of the findings.

Although the four chain mediation models constructed in this study were statistically significant, the effect sizes of some paths were relatively small, and their practical significance should be interpreted with caution. Specifically, the paths LBE→MA→ES→Dep (*B* = 0.02) and LBE→PA→ES→Dep (*B* = 0.03) were statistically significant but made limited practical contributions. This is consistent with the results of the between-group comparisons, and several statistically significant between-group differences (such as maternal attachment, cognitive reappraisal, and expressive suppression) also exhibit relatively small effect sizes. This pattern may be attributable to the influence of sample size on statistical significance, as large samples can detect effects that are negligible in magnitude [63]. Moreover, both pathways involve expressive suppression as the second mediator, suggesting that parental attachment has a relatively weak association with expressive suppression, or that the mediating role of expressive suppression between parental attachment and depression is less substantial than that of cognitive reappraisal. Thus, among left-behind children, although expressive suppression is associated with depression, its cumulative effect within chain mediation pathways is relatively small, and it may function more as a direct risk factor rather than a key mediating mechanism. Accordingly, future intervention studies should prioritize pathways involving cognitive reappraisal, which demonstrate larger effect sizes, rather than overemphasizing the indirect chain effects of expressive suppression.

Based on the findings of this study, we propose several tentative and speculative intervention directions. The observed decrease in the centrality of maternal attachment suggests that future research could examine whether training in maternal emotional expression can strengthen emotional bonds. Considering the strong association between cognitive reappraisal and depression, school-based cognitive reconstruction courses may help children reinterpret parental migration as “family striving” rather than “emotional abandonment” [64]. Moreover, the highest expected influence of paternal attachment suggests that problem-solving-oriented activities (such as robotics and physical games) could be explored to determine whether they can simulate the cognitive challenges typically provided by fathers, thereby partially compensating for the absence of paternal attachment [65]. It should be emphasized that these suggestions are speculative rather than prescriptive and are intended to inform future research directions. Rigorous evaluation in controlled trials would be required before any causal inferences can be drawn.

Furthermore, future research should integrate experimental paradigms and psychophysiological measures to enhance construct validity and mechanistic interpretation. For example, experimental emotion regulation paradigms (e.g., instructed reappraisal vs. suppression tasks using affective stimuli) could be combined with emotion‑eliciting materials (e.g., negative pictures or film clips) to obtain behavioral indices of regulation efficacy, rather than relying solely on self‑reported strategy use [66]. At the physiological level, event‑related potentials (ERPs) could index modulation of emotional reactivity during regulation attempts [67]. Pupillometry could capture cognitive effort and regulatory engagement during emotion regulation [68, 69]. Autonomic indices (e.g., heart rate variability as a proxy for regulatory flexibility) may provide insight into regulatory capacity associated with attachment‑related differences. Oscillatory EEG dynamics and functional connectivity could help examine whether attachment‑related differences are associated with altered fronto‑limbic coupling during regulation [70]. Integrating these approaches would allow future studies to move beyond statistical mediation and network topology toward a deeper understanding of the causal processes and dynamic mechanisms linking left‑behind experience, attachment, emotion regulation, and depression.

## Conclusion

This study integrated network analysis and chain mediation models to elucidate the psychological mechanisms through which left-behind experiences influence child depression via differentiated pathways. Left-behind experiences are associated with weakened parent-child attachment, reduced cognitive reappraisal, increased expressive suppression, and a heightened risk of depression. Network analysis revealed weakened maternal attachment centrality, lower maternal-paternal connectivity in the LBC group and the highest expected influence of paternal attachment in all groups. LBC with both parents absent showed network structure and global strength distinct from NLBC, indicating that the absence of both parents has a far greater effect on reshaping children’s psychological networks than the absence of the father alone. The core finding is that parent-child attachment and emotion regulation mediated the association between left-behind experiences and depression. These findings provide practical insights for identifying the risk of depression in left-behind children and developing targeted interventions.

## Supplementary Information

Supplementary Material 1.

## Data Availability

The datasets generated and analysed during the current study are available in the Open Science Framework repository, 10.17605/OSF.IO/6H7UM.

## Abbreviations

LBC: Left-behind Children
NLBC: Non-left-behind Children
IPPA: Inventory of Parent and Peer Attachment
ERQ-CA: Emotion Regulation Questionnaire for Children and Adolescents
CDI: Children’s Depression Inventory
MANOVA: Multivariate Analysis of Variance
NCT: Network Comparison Test
MA: Maternal Attachment
PA: Paternal Attachment
PeA: Peer Attachment
CR: Cognitive Reappraisal
ES: Expressive Suppression
Dep: Depression
LBE: Left-behind Experience
