# Supplementary material for: Depression in left-behind children: a network analysis and chained mediation of attachment and emotion regulation
Source: BMC Psychol. 2026 May 28;14:1099. doi: 10.1186/s40359-026-04848-0 (PMC13397604; doi:10.1186/s40359-026-04848-0)
Supplement: Supplementary file 1 — Supplementary Material 1. [file 40359_2026_4848_MOESM1_ESM.docx]

Supplementary Material

# 1 Left-behind Status Questionnaire

The Chinese version and English version of Left-behind Status Questionnaire are presented.

## *1.1 Chinese version of Left-behind Status Questionnaire*

亲爱的同学：

你好！

欢迎参加我们的问卷调查，这是一份关于你及你家庭的简单调查。问卷中问题的答案没有对错之分，请你根据自己平时的想法和实际情况，认真回答问卷中的问题。我们郑重承诺，对于你所填写的所有信息，我们将严格保密，不会泄露给任何个人和机构，非常感谢你的支持！

回答选择题时，请在选中的选项上画“√”；回答填空题时，请在横线处写上文字或数字。

**个人基本信息**

1. 性别：①男 ②女

2. 年龄： 岁

3. 民族：①汉 ②其他

4. 除了自己外，你还有几位兄弟姐妹？（包括一起生活的同父异母、同母异父的未成年的兄弟姐妹，但堂、表兄弟姐妹不算）

①0个 ②1个 ③2个 ④3个及以上

1. 你在家排行

①独生子女，无所谓排行 ②第一 ③第二 ④第三及以上

1. 户口所在地：①城市 ②农村
2. 你爸爸的受教育程度：

①没有上过学 ②小学 ③初中 ④高中或中专 ⑤大学 ⑥研究生 ⑦不清楚

1. 你妈妈的受教育程度：

①没有上过学 ②小学 ③初中 ④高中或中专 ⑤大学 ⑥研究生 ⑦不清楚

**留守经历调查**

1. 你爸爸是否有外出打工/经商的经历（6个月以上）：

①现在是 ②以前有过 ③从来没有

1. 你爸爸第一次外出打工时，你的年龄多大（实岁）
2. 你爸爸外出打工多少年：
3. 你妈妈是否有外出打工/经商的经历（6个月以上）：

①现在是 ②以前有过 ③从来没有

1. 你妈妈第一次外出打工时，你的年龄多大（实岁）
2. 你妈妈外出打工多少年：

**如果你的父亲或母亲在你上大学之前曾外出打工/经商一段时间，这段时间被称为留守时期。如果你经历过留守时期，请填写以下问题；如果没有，则不用填写：**

1. 如果你的父亲或母亲在你上大学之前曾外出打工/经商一段时间，在这些留守期间，你和谁住在一起：（可多选）

①爷爷 ②奶奶 ③外公 ④外婆 ⑤爸爸 ⑥妈妈 ⑦亲戚 （填写称呼） ⑧单独一人 ⑨兄弟姐妹 ⑩其他

1. 留守期间，照看你的人是谁：（可多选）

①爷爷 ②奶奶 ③外公 ④外婆 ⑤爸爸 ⑥妈妈 ⑦亲戚 （填写称呼）

1. 留守期间照看你的人，他（或她）的年龄是：

①小于20岁 ②20-40岁 ③40-60岁 ④60岁以上

1. 留守期间照看你的人，他（或她）的学历是：

①没有上过学 ②小学 ③初中 ④高中或中专 ⑤大学（专科或本科） ⑥研究生 ⑦不清楚

1. 留守期间，你和照看你的人的沟通频率是：

①极少或基本不沟通 ②偶尔沟通 ③经常沟通

1. 留守期间，你与外出父母的联系情况是：

①极少或基本不联系 ②偶尔联系 ③经常联系

1. 留守期间，你与外出父母最主要的联系方式是：

①文字联系（信件等） ②语音联系（电话等） ③视频联系（微信等）

④其他

1. 留守期间，父母与你联系时说得最多的是什么？

①学习问题 ②生活问题 ③安全问题 ④心理问题 ⑤其他

1. 留守期间，你的父母多久回家一次？

①1个星期 ②1个月 ③2-6个月 ④半年 ⑤1年 ⑥1-2年 ⑦2年 以上

1. 留守期间，你的父母每次回家待几天：

## *1.2 English version of Left-behind Status Questionnaire*

**Introduction**

Dear Classmate:

Hello!

Welcome to participate in our questionnaire survey. This is a simple survey about you and your family. There are no right or wrong answers to the questions in the questionnaire. Please answer the questions carefully according to your usual thoughts and actual situations. We solemnly promise that we will strictly keep confidential all the information you fill in and will not disclose it to any individual or institution. Thank you very much for your support!

When answering multiple - choice questions, please mark a “√” on the selected option. When answering fill - in - the - blank questions, please write words or numbers on the horizontal line.

**Personal Basic Information**

1. Gender: ①Male ②Female
2. Age: __________ years old
3. Ethnicity: ①Han ②Other
4. Besides yourself, how many siblings do you have? (Including minor siblings with the same father or mother, or step - siblings living together, but cousins are not included)

①0 ②1 ③2 ④3 or more

1. Your birth order in the family:

①Only child, no birth order applicable ②The eldest ③The second - born ④The third - born or younger

1. Place of household registration: ①City ②Rural area
2. Your father's educational attainment:

①Never attended school ②Primary school ③Junior high school ④Senior high school or technical secondary school ⑤University ⑥Postgraduate ⑦Unsure

1. Your mother's educational attainment:

①Never attended school ②Primary school ③Junior high school ④Senior high school or technical secondary school ⑤University ⑥Postgraduate ⑦Unsure

**Survey on Left - Behind Experience**

1. Did your father have the experience of working or doing business away from home for more than 6 months?

①Currently ②Used to ③Never

2. How old (in full years) were you when your father first went to work away from home?

3. How many years did your father work away from home?

4. Did your mother have the experience of working or doing business away from home for more than 6 months?

①Currently ②Used to ③Never

5. How old (in full years) were you when your mother first went to work away from home?

6. How many years did your mother work away from home?

**If either your father or mother worked or did business away from home for a period of time, this period is called the left - behind period. If you have experienced the left - behind period, please fill in the following questions; if not, you don't need to fill them in:**

7. If either your father or mother worked or did business away from home for a period of time, during these left - behind periods, who did you live with? (Multiple choices are available)

①Grandfather ②Grandmother ③Maternal grandfather ④Maternal grandmother ⑤Father ⑥Mother ⑦Relative (Please fill in the specific title) ⑧Alone ⑨Siblings ⑩Others

8. During the left - behind period, who took care of you? (Multiple choices are available)

①Grandfather ②Grandmother ③Maternal grandfather ④Maternal grandmother ⑤Father ⑥Mother ⑦Relative (Please fill in the specific title)

9. During the left - behind period, what was the age of the person taking care of you?

①Under 20 years old ②20 - 40 years old ③40 - 60 years old ④Over 60 years old

10. During the left - behind period, what was the educational attainment of the person taking care of you?

①Never attended school ②Primary school ③Junior high school ④Senior high school or technical secondary school ⑤University (junior college or undergraduate) ⑥Postgraduate ⑦Unsure

11. During the left - behind period, what was the frequency of your communication with the person taking care of you?

①Rarely or hardly communicate ②Occasionally communicate ③Often communicate

12. During the left - behind period, what was your contact situation with your parents away from home?

①Rarely or hardly contact ②Occasionally contact ③Often contact

13. During the left - behind period, what was the main way of communication between you and your parents away from home?

①Text - based communication (letters, etc.) ②Voice - based communication (phone calls, etc.) ③Video - based communication (WeChat video calls, etc.) ④Others

14. During the left - behind period, what did your parents talk about most when they contacted you?

①Study - related issues ②Life - related issues ③Safety - related issues ④Mental health - related issues ⑤Others

15. During the left - behind period, how often did your parents go home?

①Once a week ②Once a month ③Every 2 - 6 months ④Half a year ⑤Once a year ⑥Once every 1 - 2 years ⑦Once every 2 years or more

16. During the left - behind period, how many days did your parents stay at home each time they went home?

# 2 Parent and Peer Attachment Questionnaire

The Chinese version and English version of Parent and Peer Attachment Questionnaire are presented.

## *2.1 Chinese version of Parent and Peer Attachment Questionnaire*

**指导语**

本问卷问及生活中的重要人物，包括妈妈、爸爸和亲密朋友，与你之间的关系。请仔细阅读每个部分的说明。

| **第一部分**  下列某些句子问及你对**妈妈**的感受，或者是你对扮演妈妈角色的那个人的感受。如果有几个扮演妈妈角色的人（例如，生母、继母、奶奶、外婆、阿姨等），选择对你影响最大的那个人进行回答。  请阅读每个句子，在每题末的括号内填入**最符合**你情况的那一个数字。  **1=完全不符合 2=不太符合 3=有时符合 4=常常符合 5=完全符合** |
| --- |

|  | **完全不符合** | **不太符合** | **有时符合** | **常常符合** | **完全符合** |
| --- | --- | --- | --- | --- | --- |
| 1. 妈妈尊重我的感受。 | **1** | **2** | **3** | **4** | **5** |
| 1. 我感到，妈妈是很称职的妈妈。 | **1** | **2** | **3** | **4** | **5** |
| 1. 我希望有一个和现在不一样的妈妈。 | **1** | **2** | **3** | **4** | **5** |
| 1. 妈妈接受我的现状。 | **1** | **2** | **3** | **4** | **5** |
| 1. 对于我关切的事情，我喜欢征求妈妈的意见。 | **1** | **2** | **3** | **4** | **5** |
| 1. 我感到，将我自己的感受告诉妈妈没有用。 | **1** | **2** | **3** | **4** | **5** |
| 1. 我心烦意乱的时候，妈妈能觉察到。 | **1** | **2** | **3** | **4** | **5** |
| 1. 如果和妈妈讨论我的难题，我感到害羞或者感到愚蠢。 | **1** | **2** | **3** | **4** | **5** |
| 1. 妈妈对我期望太多。 | **1** | **2** | **3** | **4** | **5** |
| 1. 和妈妈相处，我感到心烦意乱。 | **1** | **2** | **3** | **4** | **5** |
| 1. 我感受到的烦恼比妈妈所知道的要多得多。 | **1** | **2** | **3** | **4** | **5** |
| 1. 当妈妈和我讨论事情时，妈妈在意我的观点。 | **1** | **2** | **3** | **4** | **5** |
| 1. 妈妈信任我的判断。 | **1** | **2** | **3** | **4** | **5** |
| 1. 妈妈有自己的难题，因此，我有难题时也不打扰他。 | **1** | **2** | **3** | **4** | **5** |
| 1. 妈妈帮助我更好地理解我自己。 | **1** | **2** | **3** | **4** | **5** |
| 1. 我将我的难题和烦恼告诉妈妈。 | **1** | **2** | **3** | **4** | **5** |
| 1. 我对妈妈感到愤怒。 | **1** | **2** | **3** | **4** | **5** |
| 1. 我从妈妈那里没有得到什么关注。 | **1** | **2** | **3** | **4** | **5** |
| 1. 妈妈帮助我讨论我的困难。 | **1** | **2** | **3** | **4** | **5** |
| 1. 妈妈理解我。 | **1** | **2** | **3** | **4** | **5** |
| 1. 当我对某事愤怒时，妈妈试图理解我。 | **1** | **2** | **3** | **4** | **5** |
| 1. 我信任妈妈。 | **1** | **2** | **3** | **4** | **5** |
| 1. 妈妈不了解我最近的状况。 | **1** | **2** | **3** | **4** | **5** |
| 1. 我可以依赖妈妈，摆脱情绪困扰。 | **1** | **2** | **3** | **4** | **5** |
| 25. 如果妈妈知道某事困扰我，他向我询问有关情况。 | **1** | **2** | **3** | **4** | **5** |

| **第二部分**  这部分问及你对**爸爸**的感受，或者是你对扮演爸爸角色的那个人的感受。如果有几个扮演爸爸角色的人（例如，亲生爸爸、继父、爷爷、外公、叔叔等），选择对你影响最大的那个人进行回答。  请阅读每个句子，在每题末的括号内填入**最符合**你情况的那一个数字。  **1=完全不符合 2=不太符合 3=有时符合 4=常常符合 5=完全符合** |
| --- |

|  | **完全不符合** | **不太符合** | **有时符合** | **常常符合** | **完全符合** |
| --- | --- | --- | --- | --- | --- |
| 1. 爸爸尊重我的感受。 | **1** | **2** | **3** | **4** | **5** |
| 1. 我感到，爸爸是很称职的爸爸。 | **1** | **2** | **3** | **4** | **5** |
| 1. 我希望有一个和现在不一样的爸爸。 | **1** | **2** | **3** | **4** | **5** |
| 1. 爸爸接受我的现状。 | **1** | **2** | **3** | **4** | **5** |
| 1. 对于我关切的事情，我喜欢征求爸爸的意见。 | **1** | **2** | **3** | **4** | **5** |
| 1. 我感到，将我自己的感受告诉爸爸没有用。 | **1** | **2** | **3** | **4** | **5** |
| 1. 我心烦意乱的时候，爸爸能觉察到。 | **1** | **2** | **3** | **4** | **5** |
| 1. 如果和爸爸讨论我的难题，我感到害羞或者感到愚蠢。 | **1** | **2** | **3** | **4** | **5** |
| 1. 爸爸对我期望太多。 | **1** | **2** | **3** | **4** | **5** |
| 1. 和爸爸相处，我感到心烦意乱。 | **1** | **2** | **3** | **4** | **5** |
| 1. 我感受到的烦恼比爸爸所知道的要多得多。 | **1** | **2** | **3** | **4** | **5** |
| 1. 当爸爸和我讨论事情时，爸爸在意我的观点。 | **1** | **2** | **3** | **4** | **5** |
| 1. 爸爸信任我的判断。 | **1** | **2** | **3** | **4** | **5** |
| 1. 爸爸有自己的难题，因此，我有难题时也不打扰他。 | **1** | **2** | **3** | **4** | **5** |
| 1. 爸爸帮助我更好地理解我自己。 | **1** | **2** | **3** | **4** | **5** |
| 1. 我将我的难题和烦恼告诉爸爸。 | **1** | **2** | **3** | **4** | **5** |
| 1. 我对爸爸感到愤怒。 | **1** | **2** | **3** | **4** | **5** |
| 1. 我从爸爸那里没有得到什么关注。 | **1** | **2** | **3** | **4** | **5** |
| 1. 爸爸帮助我讨论我的困难。 | **1** | **2** | **3** | **4** | **5** |
| 1. 爸爸理解我。 | **1** | **2** | **3** | **4** | **5** |
| 1. 当我对某事愤怒时，爸爸试图理解我。 | **1** | **2** | **3** | **4** | **5** |
| 1. 我信任爸爸。 | **1** | **2** | **3** | **4** | **5** |
| 1. 爸爸不了解我最近的状况。 | **1** | **2** | **3** | **4** | **5** |
| 1. 我可以依赖爸爸，摆脱情绪困扰。 | **1** | **2** | **3** | **4** | **5** |
| 1. 如果爸爸知道某事困扰我，他向我询问有关情况。 | **1** | **2** | **3** | **4** | **5** |

| **第三部分**  这个部分问及你与**亲密朋友们**之间的关系。  请阅读每个句子，在每题末的括号内填入**最符合**你情况的那一个数字。  **1=完全不符合2=不太符合3=有时符合4=常常符合5=完全符合** |
| --- |

|  | **完全不符合** | **不太符合** | **有时符合** | **常常符合** | **完全符合** |
| --- | --- | --- | --- | --- | --- |
| 1. 对于我关切的事情，我喜欢征求朋友们的意见。 | **1** | **2** | **3** | **4** | **5** |
| 1. 我心烦意乱的时候，朋友们能理解我。 | **1** | **2** | **3** | **4** | **5** |
| 1. 当我们讨论事情时，朋友们在意我的观点。 | **1** | **2** | **3** | **4** | **5** |
| 1. 和朋友们讨论我的难题使我感到害羞或者感到愚蠢。 | **1** | **2** | **3** | **4** | **5** |
| 1. 我希望更换新的不同的朋友。 | **1** | **2** | **3** | **4** | **5** |
| 1. 朋友们理解我。 | **1** | **2** | **3** | **4** | **5** |
| 1. 朋友们鼓励我说出我自己的困难。 | **1** | **2** | **3** | **4** | **5** |
| 1. 朋友们接受我的现状。 | **1** | **2** | **3** | **4** | **5** |
| 1. 我感到有必要与朋友们更频繁地接触。 | **1** | **2** | **3** | **4** | **5** |
| 1. 朋友们不理解我最近的状况。 | **1** | **2** | **3** | **4** | **5** |
| 1. 当我和朋友们在一起的时候，我感到孤单或者感到有距离。 | **1** | **2** | **3** | **4** | **5** |
| 1. 朋友们仔细听我说话。 | **1** | **2** | **3** | **4** | **5** |
| 1. 我感到我的朋友们都很好。 | **1** | **2** | **3** | **4** | **5** |
| 1. 要和朋友们说上话很容易。 | **1** | **2** | **3** | **4** | **5** |
| 1. 当我对某事愤怒时，朋友们试图理解我。 | **1** | **2** | **3** | **4** | **5** |
| 1. 朋友们帮助我更好地理解我自己。 | **1** | **2** | **3** | **4** | **5** |
| 1. 朋友们在意我的感受。 | **1** | **2** | **3** | **4** | **5** |
| 1. 我对朋友们感到愤怒。 | **1** | **2** | **3** | **4** | **5** |
| 1. 我可以依赖朋友们，摆脱情绪困挠。 | **1** | **2** | **3** | **4** | **5** |
| 1. 我信任朋友们。 | **1** | **2** | **3** | **4** | **5** |
| 1. 朋友们尊重我的感受。 | **1** | **2** | **3** | **4** | **5** |
| 1. 我感受到的烦恼比朋友们所知道的要多得多。 | **1** | **2** | **3** | **4** | **5** |
| 1. 朋友们似乎毫无理由地激怒我。 | **1** | **2** | **3** | **4** | **5** |
| 1. 我可以将我的难题和烦恼告诉朋友们。 | **1** | **2** | **3** | **4** | **5** |
| 1. 如果朋友们知道了某事困扰我，他们向我询问有关情况。 | **1** | **2** | **3** | **4** | **5** |

## *2.2 English version of Parent and Peer Attachment Questionnaire*

**Instruction**

This questionnaire asks about your relationships with important people in your life, including your mother, father, and close friends. Please read the instructions for each section carefully.

| **Section 1**  This questionnaire asks about your relationships with your **mother**. Each of the following statements asks about your feelings about your mother or the woman who has acted as your mother (e.g., a natural mother and a step-mother). Answer the questions for the one you feel has most influenced you.  Please read each statement carefully and choose the number that best describes your situation by filling it in the parentheses at the end of each item.  **1 = Almost Never 2 = Not Very 3 = Sometimes 4 = Often 5 = Almost Always** |
| --- |

|  | **Almost Never** | **Not Very** | **Sometimes** | **Often** | **Almost Always** |
| --- | --- | --- | --- | --- | --- |
| 1. My mother respects my feelings | **1** | **2** | **3** | **4** | **5** |
| 1. I feel my mother does a good job as my mother | **1** | **2** | **3** | **4** | **5** |
| 1. I wish I had a different mother | **1** | **2** | **3** | **4** | **5** |
| 1. My mother accepts me as I am | **1** | **2** | **3** | **4** | **5** |
| 1. I like to get my mother’s point of view on things I’m concerned about | **1** | **2** | **3** | **4** | **5** |
| 1. I feel it’s no use letting my feelings show around my mother | **1** | **2** | **3** | **4** | **5** |
| 1. My mother can tell when I’m upset about something | **1** | **2** | **3** | **4** | **5** |
| 1. Talking over my problems with my mother makes me feel ashamed or foolish | **1** | **2** | **3** | **4** | **5** |
| 1. My mother expects too much from me | **1** | **2** | **3** | **4** | **5** |
| 1. I get upset easily around my mother | **1** | **2** | **3** | **4** | **5** |
| 1. I get upset a lot more than my mother knows about | **1** | **2** | **3** | **4** | **5** |
| 1. When we discuss things, my mother cares about my point of view | **1** | **2** | **3** | **4** | **5** |
| 1. My mother trusts my judgment | **1** | **2** | **3** | **4** | **5** |
| 1. My mother has her own problems, so I don’t bother her with mine | **1** | **2** | **3** | **4** | **5** |
| 1. My mother helps me understand myself better | **1** | **2** | **3** | **4** | **5** |
| 1. I tell my mother about my problems and troubles | **1** | **2** | **3** | **4** | **5** |
| 1. I feel angry with my mother | **1** | **2** | **3** | **4** | **5** |
| 1. I don’t get much attention from my mother | **1** | **2** | **3** | **4** | **5** |
| 1. My mother helps me talk about my difficulties | **1** | **2** | **3** | **4** | **5** |
| 1. My mother understands me | **1** | **2** | **3** | **4** | **5** |
| 1. When I am angry about something, my mother tries to be understanding | **1** | **2** | **3** | **4** | **5** |
| 1. I trust my mother | **1** | **2** | **3** | **4** | **5** |
| 1. My mother doesn’t understand what I’m going through these days | **1** | **2** | **3** | **4** | **5** |
| 1. I can count on my mother when I need to get something off my chest | **1** | **2** | **3** | **4** | **5** |
| 1. If my mother knows something is bothering me, she asks me about it | **1** | **2** | **3** | **4** | **5** |

| **Section 2**  This next section of questionnaire asks about your relationships with your **father**. Each of the following statements asks about your feelings about your father or the man who has acted as your father (e.g., a natural father and a step-father). Answer the questions for the one you feel has most influenced you.  Please read each statement carefully and choose the number that best describes your situation by filling it in the parentheses at the end of each item.  **1 = Almost Never 2 = Not Very 3 = Sometimes 4 = Often 5 = Almost Always** |
| --- |

|  | **Almost Never** | **Not Very** | **Sometimes** | **Often** | **Almost Always** |
| --- | --- | --- | --- | --- | --- |
| 1. My father respects my feelings. | **1** | **2** | **3** | **4** | **5** |
| 1. I feel my father does a good job as my father. | **1** | **2** | **3** | **4** | **5** |
| 1. I wish I had a different father. | **1** | **2** | **3** | **4** | **5** |
| 1. My father accepts me as I am. | **1** | **2** | **3** | **4** | **5** |
| 1. I like to get my father’s point of view on things I’m concerned about. | **1** | **2** | **3** | **4** | **5** |
| 1. I feel it’s no use letting my feelings show around my father. | **1** | **2** | **3** | **4** | **5** |
| 1. My father can tell when I’m upset about something. | **1** | **2** | **3** | **4** | **5** |
| 1. Talking over my problems with my father | **1** | **2** | **3** | **4** | **5** |
| 1. My father expects too much from me. | **1** | **2** | **3** | **4** | **5** |
| 1. I get upset easily around my father. | **1** | **2** | **3** | **4** | **5** |
| 1. I get upset a lot more than my father knows about. | **1** | **2** | **3** | **4** | **5** |
| 1. When we discuss things, my father cares about my point of view. | **1** | **2** | **3** | **4** | **5** |
| 1. My father trusts my judgment. | **1** | **2** | **3** | **4** | **5** |
| 1. My father has her own problems, so I don’t bother her with mine. | **1** | **2** | **3** | **4** | **5** |
| 1. My father helps me understand myself better. | **1** | **2** | **3** | **4** | **5** |
| 1. I tell my father about my problems and troubles. | **1** | **2** | **3** | **4** | **5** |
| 1. I feel angry with my father. | **1** | **2** | **3** | **4** | **5** |
| 1. I don’t get much attention from my father. | **1** | **2** | **3** | **4** | **5** |
| 1. My father helps me talk about my difficulties. | **1** | **2** | **3** | **4** | **5** |
| 1. My father understands me. | **1** | **2** | **3** | **4** | **5** |
| 1. When I am angry about something, my father tries to be understanding. | **1** | **2** | **3** | **4** | **5** |
| 1. I trust my father. | **1** | **2** | **3** | **4** | **5** |
| 1. My father doesn’t understand what I’m going through these days. | **1** | **2** | **3** | **4** | **5** |
| 1. I can count on my father when I need to get something off my chest. | **1** | **2** | **3** | **4** | **5** |
| 1. If my father knows something is bothering me, she asks me about it. | **1** | **2** | **3** | **4** | **5** |

| **Section 3**  This next section of questionnaire asks about your relationships with your **close friends**.  Please read each statement carefully and choose the number that best describes your situation by filling it in the parentheses at the end of each item.  **1 = Almost Never 2 = Not Very 3 = Sometimes 4 = Often 5 = Almost Always** |
| --- |

|  | **Almost Never** | **Not Very** | **Sometimes** | **Often** | **Almost Always** |
| --- | --- | --- | --- | --- | --- |
| 1. I like to get my friend’s point of view on things I’m concerned about. | **1** | **2** | **3** | **4** | **5** |
| 1. My friends can tell when I’m upset about something. | **1** | **2** | **3** | **4** | **5** |
| 1. When we discuss things, my friends care about my point of view. | **1** | **2** | **3** | **4** | **5** |
| 1. When I discuss things, my friends care about my point of view. | **1** | **2** | **3** | **4** | **5** |
| 1. I wish I had different friends. | **1** | **2** | **3** | **4** | **5** |
| 1. My friends understand me. | **1** | **2** | **3** | **4** | **5** |
| 1. My friends help me to talk about my difficulties. | **1** | **2** | **3** | **4** | **5** |
| 1. My friends accept me as I am. | **1** | **2** | **3** | **4** | **5** |
| 1. I feel the need to be in touch with my friends more often. | **1** | **2** | **3** | **4** | **5** |
| 1. My friends don’t understand what I’m going through these days. | **1** | **2** | **3** | **4** | **5** |
| 1. I feel alone or apart when I’m with my friends. | **1** | **2** | **3** | **4** | **5** |
| 1. My friends listen to what I have to say. | **1** | **2** | **3** | **4** | **5** |
| 1. I feel my friends are good friends. | **1** | **2** | **3** | **4** | **5** |
| 1. My friends are fairly easy to talk to. | **1** | **2** | **3** | **4** | **5** |
| 1. When I am angry about something, my friends try to be understanding. | **1** | **2** | **3** | **4** | **5** |
| 1. My friends help me to understand myself better. | **1** | **2** | **3** | **4** | **5** |
| 1. My friends care about how I am. | **1** | **2** | **3** | **4** | **5** |
| 1. I feel angry with my friends. | **1** | **2** | **3** | **4** | **5** |
| 1. I can count on my friends when I need to get something off my chest. | **1** | **2** | **3** | **4** | **5** |
| 1. I trust my friends. | **1** | **2** | **3** | **4** | **5** |
| 1. My friends respect my feelings. | **1** | **2** | **3** | **4** | **5** |
| 1. I get upset a lot more than my friends know about. | **1** | **2** | **3** | **4** | **5** |
| 1. It seems as if my friends are irritated with me for no reason. | **1** | **2** | **3** | **4** | **5** |
| 1. I can tell my friends about my problems and troubles. | **1** | **2** | **3** | **4** | **5** |
| 1. If my friends know something is bothering me, they ask me about it. | **1** | **2** | **3** | **4** | **5** |

# 3 Emotion Regulation Questionnaire for Children and Adolescents

## *3.1 Chinese version of Emotion Regulation Questionnaire* *for Children and Adolescents*

| 以下的表述关于你的情绪生活。请仔细阅读，并且在每一项表述中用打勾(√)的方式表明你赞同或者不赞同的水平。答案无对错，请你根据实际情况作答。  分数分为5个等级：1.完全不同意；2.有点不同意；3.中性；4.有点同意；5.完全同意。 | | | | | |
| --- | --- | --- | --- | --- | --- |
|  | **完全**  **不同意** | **有点**  **不同意** | **中性** | **有点**  **同意** | **完全**  **同意** |
| 1. 当我想要高兴的时候，我会想些其他的事情（如今天老师表扬了我）。 | **1** | **2** | **3** | **4** | **5** |
| 2. 我不愿意表露自己的情绪。 | **1** | **2** | **3** | **4** | **5** |
| 3. 当我不高兴（如伤心、生气或担心）的时候，我会想一些让自己高兴的事情。 | **1** | **2** | **3** | **4** | **5** |
| 4. 当我高兴的时候，我会小心地不让它们表现出来。 | **1** | **2** | **3** | **4** | **5** |
| 5. 当我担心某件事情时，我会从积极的角度去想它，从而使心情感觉良好。 | **1** | **2** | **3** | **4** | **5** |
| 6. 我通过不表现情绪的方式来控制自己的情绪。 | **1** | **2** | **3** | **4** | **5** |
| 7. 当我在某件事情中想感受到更多快乐时，我会换个角度想事情。 | **1** | **2** | **3** | **4** | **5** |
| 8. 我会换个角度想问题来控制自己的情绪。 | **1** | **2** | **3** | **4** | **5** |
| 9. 当我不高兴（如伤心、生气或担心）的时候，我不会让情绪表现出来。 | **1** | **2** | **3** | **4** | **5** |
| 10. 当我遇到不高兴的事情时，我会从积极的方面想事情，从而使心情感觉良好。 | **1** | **2** | **3** | **4** | **5** |

## *3.2 English version of Emotion Regulation Questionnaire* *for Children and Adolescents*

| The following statements pertain to your emotional life. Please read carefully and indicate your level of agreement or disagreement with each statement by marking (√). There are no right or wrong answers; please respond according to your actual situation.  The scoring is divided into five levels: 1. Strongly Disagree; 2. Somewhat Disagree; 3. Neutral; 4. Somewhat Agree; 5. Strongly Agree. | | | | | |
| --- | --- | --- | --- | --- | --- |
|  | **Almost Never** | **Slightly disagree** | **Neutral** | **Slightly agree** | **Almost Always** |
| 1. When I want to be happy, I think about other things (like the teacher praised me today). | **1** | **2** | **3** | **2** | **5** |
| 2. I don’t want to show my . | **1** | **2** | **3** | **4** | **5** |
| 3. When I’m upset (like sad, angry, or worried), I think about things that make me happy. | **1** | **2** | **3** | **4** | **5** |
| 4. When I’m happy, I'm careful not to let them show. | **1** | **2** | **3** | **4** | **5** |
| 5. When I’m worried about something, I think about it in a positive light, which makes me feel good. | **1** | **2** | **3** | **4** | **5** |
| 6. I control my emotions by not showing them. | **1** | **2** | **3** | **4** | **5** |
| 7. When I want to feel more joy about something, I try to look at it from a different angle. | **1** | **2** | **3** | **4** | **5** |
| 8. I would think differently to control my emotions. | **1** | **2** | **3** | **4** | **5** |
| 9. When I’m upset (like sad, angry, or worried), I don’t let my emotions show. | **1** | **2** | **3** | **4** | **5** |
| 10. When I encounter something that I am not happy about, I think about it from the positive side so that I feel good. | **1** | **2** | **3** | **4** | **5** |

# 4 Children’s Depression Inventory

## *4.1 Chinese version of Children’s Depression Inventory*

下面的问题是了解你在**过去两周内**的实际感受。这只是一个调查，不是考试，答案没有正确或者错误之分，每个人是不一样的。请根据自己的感觉和想法在你认为符合自己的描述前**打勾(√)**。**请读完每组三句才进行这一组叙述的选择。**

| 答题举例：  **(1)** **0** 我喜欢喝茶。  **1** 我比较喜欢喝茶。  **2** 我不太喜欢喝茶。 |  |
| --- | --- |
|  |  |
| **(1) 0** 我偶尔感到悲伤。  **1** 我经常感到悲伤。  **2** 我总是感到悲伤。 | **(2)** **0** 我的将来不会有什么好的事情发生。  **1** 我不能肯定我的将来会有什么好事情发生。  **2** 我的将来会有好的事情发生。 |
| **(3)** **0** 绝大多数事情我都做得不错。  **1** 许多事情我都做错了。  **2** 我做的每一件事情都是错的。 | **(4)** **0** 我的生活中有许多让我开心的事情。  **1** 我的生活中有一些让我开心的事情。  **2** 没有什么事情是让我开心的。 |
| **(5)** **0** 我总是做不好事情。  **1** 我许多时候都做不好事情。  **2** 我偶尔做不好事情。 | **(6)** **0** 我偶尔会想到不好的事情会发生在我身上。  **1** 我担心不好的事情将发生在我身上  **2** 我确信有可怕的事情将发生在我身上。 |
| **(7)** **0** 我恨我自己。  **1** 我不喜欢我自己。  **2** 我喜欢自己。 | **(8)** **0** 所有的坏事情都是我的错。  **1** 许多坏事情是我的错。  **2** 坏事情的发生通常不是我的错。 |
| **(9)** **0** 我从没有想过自杀。  **1** 我有自杀的想法，但我不会去做。  **2** 我想自杀。 | **(10)** **0** 每天我都想哭。  **1** 许多时候我想哭。  **2** 偶尔我想哭。 |
| **(11)** **0** 总是有事情烦我。  **1** 许多时候有事情烦我。  **2** 偶尔有事情烦我。 | **(12)** **0** 我喜欢与别人在一起。  **1** 许多时候我不喜欢与别人在一起。  **2** 我总是不喜欢与别人在一起。 |
| **(13)** **0** 我不能做出决定。  **1** 我难以做出决定。  **2** 我很容易做出决定。 | **(14)** **0** 我长得不错。  **1** 我有些地方长得不好。  **2** 我长得丑陋。 |
| **(15)** **0** 我总是要逼自己去做我的功课。  **1** 许多时候我要逼自己去做我的功课。  **2** 对我来说，学校的功课不是大问题。 | 1. **0** 每天晚上我都睡不安。   **1** 许多晚上我都睡不好。  **2** 我的睡眠很好。 |
| 1. **0** 偶尔我感到疲乏。   **1** 许多时候我感到疲乏。  **2** 我总是感到疲乏。 | **(18)** **0** 大多数日子里我的食欲不好。  **1** 许多时候我的食欲不好。  **2** 许多时候我的食欲很好。 |
| **(19)** **0** 我不担心疼痛。  **1** 许多时候我担心疼痛。  **2** 我总是担心疼痛。 | **(20)** **0** 我不感到孤单。  **1** 许多时候我感到孤单。  **2** 我总是感到孤单。 |
| **(21)** **0** 学校里没有事情让我开心。  **1** 在学校里有时有事情让我感到开心。  **2** 在学校里许多时候我感到开心。 | **(22)** **0** 我有许多朋友。  **1** 我有一些朋友，但我希望有更多。  **2** 我没有任何朋友。 |
| **(23)** **0** 我的功课不错。  **1** 我的功课不如过去了。  **2** 我以前很好的科目现在却很糟糕。 | **(24)** **0** 我从来就不如别的小孩。  **1** 如果我想好的话，我就能像别的孩子一样好。  **2** 我同别的孩子一样好。 |
| **(25)** **0** 没有人真正爱我。  **1** 我不能确定是否有人爱我。  **2** 我确定有人爱我。 | **(26)** **0** 我常常做别人要我做的事。  **1** 大多数情况下，我不会做别人要我做的事。  **2** 我从不做别人要我做的事。 |
| **(27)** **0** 我能与人相处得不错。  **1** 许多时候我与人有冲突。  **2** 我总是与人冲突。 |  |

## *4.2 English version of Children’s Depression Inventory*

| The following questions aim to understand your actual feelings **over the past two weeks**. This is merely a survey, not an exam; there are no right or wrong answers, as everyone is different. Please **check (√)** the descriptions that you feel apply to you based on your feelings and thoughts. **Kindly read through each set of three statements before making your selections for that set.** | |
| --- | --- |
| Example of answering questions:  **(1) 0** I like to drink tea.  **1** I prefer to drink tea.  **2** I'm not a big fan of tea. |  |
|  |  |
| **(1)** **0** I feel sad occasionally.  **1** I often feel sad.  **2** I always feel sad. | **(2)** **0** Nothing good will happen in my future.  **.1** I’m not sure what good things will happen to me in the future.  **2** Good things will happen to me in the future. |
| **(3)** **0** I’ve done most things well.  **1** I did a lot of things wrong.  **2** Everything I did was wrong. | **(4)** **0** There are many things in my life that make me happy.  **1** There are some things in my life that make me happy.  **2** Nothing makes me happy. |
| **(5)** **0** I always don’t do things well.  **1** I don’t do things well a lot of the time.  **2** I don’t do good things once in a while. | **(6)** **0** I occasionally think that something bad will happen to me.  **1** I’m worried that something bad will happen to me.  **2** I am sure that something terrible will happen to me. |
| **(7)** **0** I hate myself.  **1** I don’t like myself.  **2** I like myself. | **(8)** **0** All the bad things are my fault.  **1** Many bad things are my fault.  **2** It’s usually not my fault that bad things happen. |
| **(9)** **0** I never thought about suicide.  **1** I have suicidal thoughts, but I’m not going to do it.  **2** I want to kill myself. | **(10)** **0** Every day I want to cry.  **1** Many times I want to cry.  **2** Occasionally I want to cry. |
| **(11)** **0** There’s always something bothering me.  **1** Many times things bother me.  **2** Occasionally, something bothers me. | **(12)** **0** I love being around people.  **1** Many times I don't like to be around people.  **2** I always don't like to be around other people. |
| **(13)** **0** I can’t make a decision.  **1** I have trouble making decisions.  **2** It was easy for me to make a decision. | **(14)** **0** I'm good-looking.  **1** There are some places where I don’t grow well.  **2** I am ugly. |
| **(15)** **0** I always have to push myself to do my homework.  **1** Many times I have to force myself to do my homework.  **2** Schoolwork is not a big problem for me. | **(16) 0** Every night I couldn’t sleep.  **1** I didn't sleep well manynights.  **2** I slept well. |
| **(17) 0** Occasionally I feel tired.  **1** Many times I feel tired.  **2** I always feel tired. | **(18)** **0** Most days I don’t have a good appetite.  **1** Many times my appetite is not good.  **2** Many times my appetite is good. |
| **(19)** **0** I’m not worried about pain.  **1** Many times I worry about pain.  **2** I’m always worried about pain. | **(20)** **0** I don’t feel alone.  **1** Many times I feel lonely.  **2** I always felt lonely. |
| **(21)** **0** There is nothing in school that makes me happy.  **1** Sometimes there are things that make me happy at school.  **2** Many times in school I feel happy. | **(22)** **0** I have many friends.  **1** I have some friends, but I wish there were more.  **2** I don’t have any friends. |
| **(23)** **0** My homework was good.  **1** My homework is not as good as it used to be.  **2** The subjects that I used to be good in are now terrible. | **(24)** **0** I’ve never been as good as other kids.  **1** If I want to be good, I can be as good as any other child.  **2** I am as good as any other child. |
| **(25)** **0** No one really loves me.  **1** I’m not sure if anyone loves me.  **2** I’m sure someone loves me. | **(26)** **0** I often do what people ask me to do.  **1** Most of the time, I don’t do what others ask me to do.  **2** I never do what others ask me to do. |
| **(27)** **0** I can get along well with people.  **1** There have been many times when I have conflicts with people.  **2** I’m always in conflict with people. |  |

# 5 Supplementary results

## *5.1 Flowchart of sample selection*


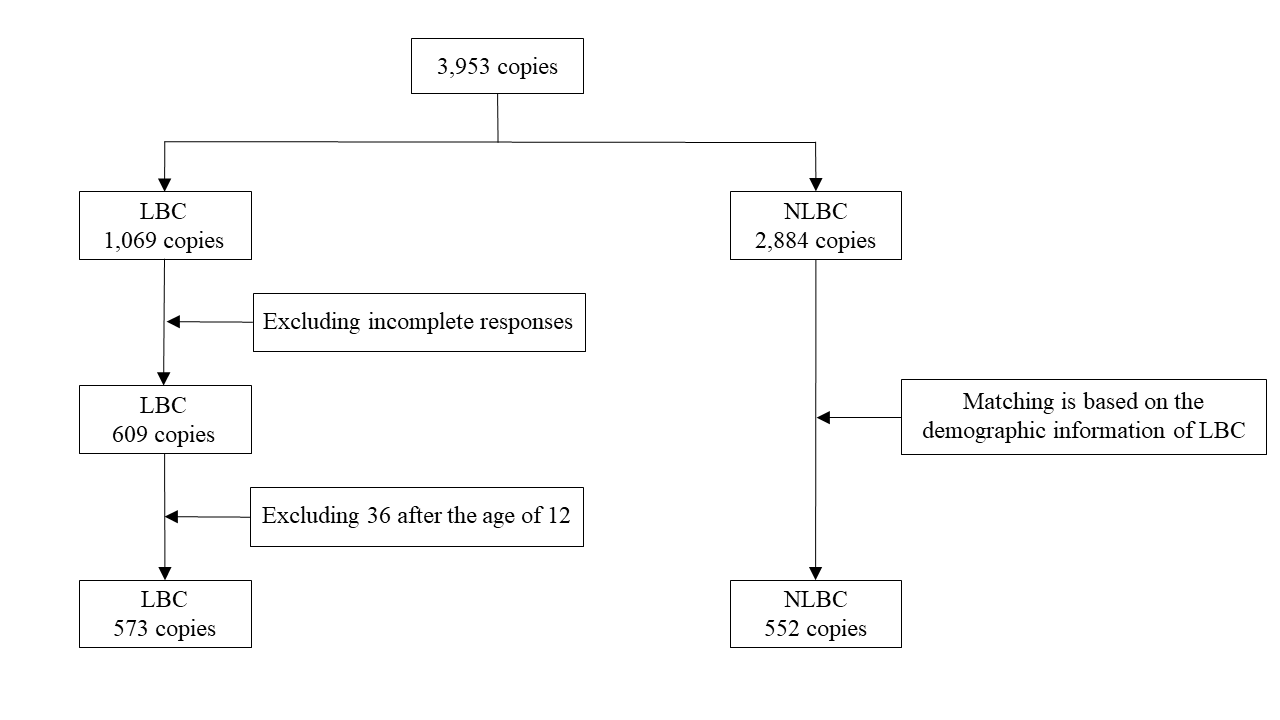


**Figure S1. Flowchart of sample selection.**

## *5.2 Demographic information between the two groups*

The results of the chi-square test indicated that there were no significant differences in demographic information between the left-behind children and the non-left-behind children.

**Table S1.** Demographic data of non-left-behind and left-behind groups.

| **Variables** | **NLBC（n=552）** | **LBC（n=573）** | ***χ^2^*** | ***p*** |
| --- | --- | --- | --- | --- |
|  | **N** | **N** |  |  |
| Gender |  |  | .06 | .804 |
| Male | 257 | 271 |  |  |
| Female | 295 | 302 |  |  |
| Nation |  |  | .80 | .372 |
| Han | 493 | 502 |  |  |
| Other | 59 | 71 |  |  |
| Siblings |  |  | 4.90 | .298 |
| 0 | 374 | 404 |  |  |
| 1 | 153 | 149 |  |  |
| 2~ | 25 | 20 |  |  |
| Order of birth |  |  | 7.21 | .205 |
| Only child | 379 | 406 |  |  |
| First | 92 | 104 |  |  |
| Second and above | 81 | 63 |  |  |
| Census register |  |  | .36 | .546 |
| Urban | 383 | 388 |  |  |
| Rural | 169 | 185 |  |  |
| Father’s level of education |  |  | 8.55 | .200 |
| Primary | 30 | 33 |  |  |
| Junior | 178 | 199 |  |  |
| Senior | 125 | 148 |  |  |
| College | 141 | 112 |  |  |
| Graduate | 19 | 12 |  |  |
| Unclear | 59 | 69 |  |  |
| Mother’s level of education |  |  | 9.62 | .142 |
| Primary | 32 | 28 |  |  |
| Junior | 169 | 212 |  |  |
| Senior | 123 | 117 |  |  |
| College | 145 | 135 |  |  |
| Graduate | 23 | 14 |  |  |
| Unclear | 60 | 67 |  |  |
| Age |  |  | 8.17 | .147 |
| 12 | 43 | 52 |  |  |
| 13 | 152 | 126 |  |  |
| 14 | 169 | 170 |  |  |
| 15 | 85 | 112 |  |  |
| 16 | 66 | 80 |  |  |
| 17 | 37 | 33 |  |  |

## *5.3 The steps of network analysis*

The steps of network analysis are as follows: (1) Network estimation. We used attachment, emotional regulation, and depression as nodes, with regularized partial correlations as edges connecting them, leading to the estimation of a regularized partial correlation network. (2) centrality estimation. To further identify key variables in the network, we used the “Least Absolute Shrinkage and Selection Operator (LASSO)” network analysis method to determine the centrality metrics of each variable. The centrality metrics of the network include strength, betweenness, closeness, and expected influence (Borsboom & Cramer, 2013). A higher value of a centrality metric indicates greater importance of that node within the network (Hu et al., 2023) (3) Testing the stability and accuracy of the network. This study employed the Bootstrap method to assess the robustness of the network structure through repeated sampling (500 times), and calculated the confidence intervals for each node and edge, visually demonstrating the stability of the network structure. Furthermore, the differences between nodes and edges were examined by plotting difference graphs to evaluate the significant differences among various indicators. (4) Network comparison. We utilized the *Network Comparison Test* package to compare the differences among the three networks. To test the differences in global and local connectivity between NLBC groups, father absent group, and father & mother absent group networks, the network comparison test (NCT) was used to test the invariance of the network structure, global strength, and node centrality by using permutation tests (1000 times) (van Borkulo et al., 2015). We also analyzed the differences between specific edges to obtain a more detailed analysis between the groups.

## *5.4 Stability and accuracy of the network*

The correlation stability (CS) coefficient was used to quantify the stability of centrality indices and represents the maximum proportion of cases that can be dropped to retain a correlation of .70 in at least 95% of the sample. The CS coefficient should not be below .25 and preferably above .50 (Zhou et al., 2022). The CS coefficients of each centrality index are shown in Table S2. The results of centrality indices stability, edge-weight accuracy, and the bootstrapped difference tests for node centrality and edge weights were shown in Figure S2–5.

**Table S2.** CS coefficients of various centrality indicators.

|  | **Strength** | **Closeness** | **Betweenness** | **EI** |
| --- | --- | --- | --- | --- |
| NLBC | .75 | .44 | .05 | .75 |
| LBC | .60 | .52 | .05 | .75 |
| father absent | .21 | .13 | .00 | .75 |
| father & mother absent group | .28 | .21 | .00 | .59 |

**Note:** EI, expected influence.


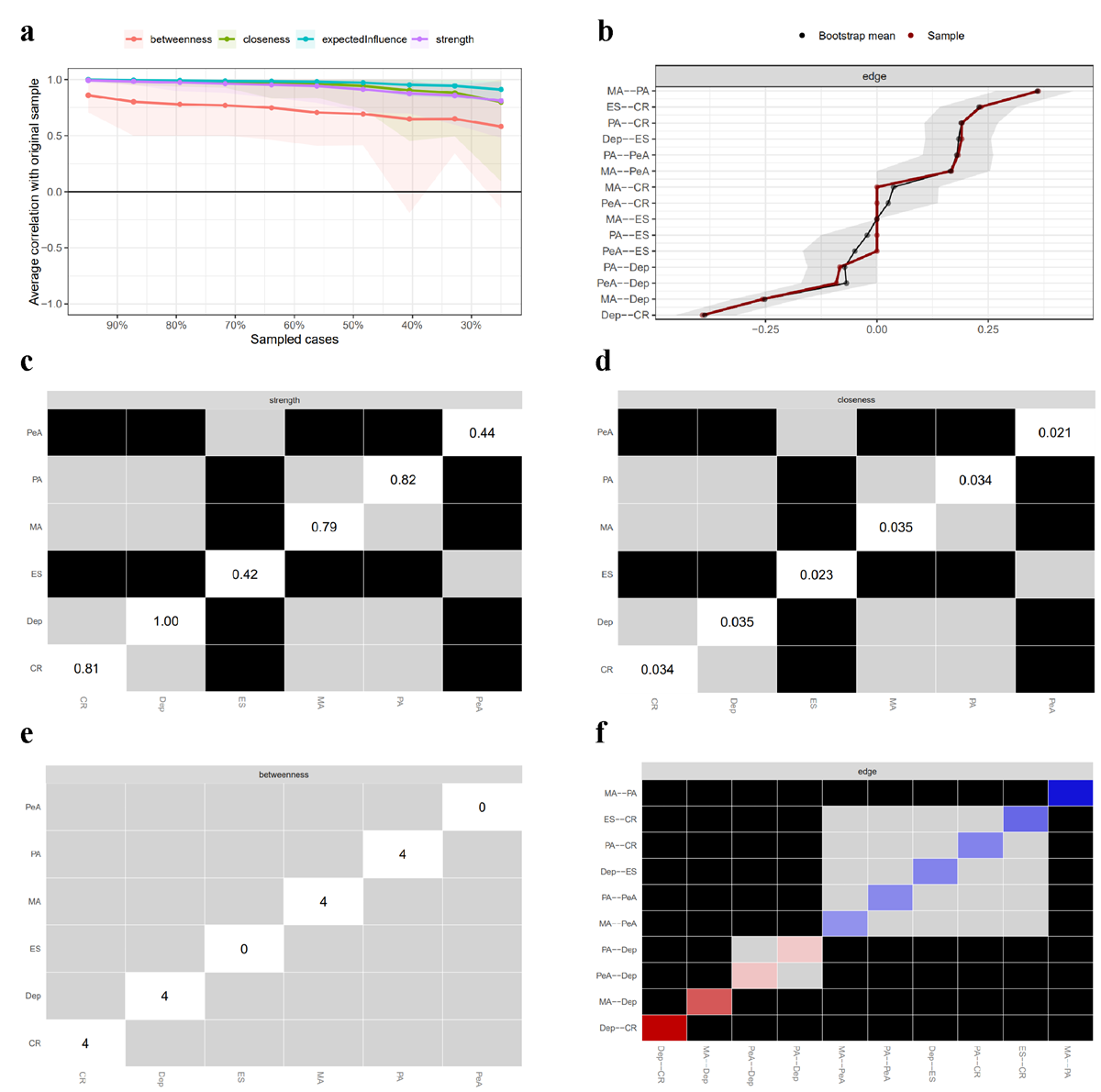


**Figure S2.** Stability and accuracy of the network for left-behind children. **a**: Different colored lines represent the average correlation between strength, closeness, betweenness, or EI within the complete sample and the subsample, respectively. The red area indicates the range from the 2.5th percentile to the 97.5th percentile; **b**: Bootstrapped confidence intervals of estimated edge-weights for left-behind children network. The red line indicates the values of edge-weight estimated in the whole sample, the grey area surrounding the red line represents the 95% quantile of the bootstrapped sampling distribution, and each horizontal line represents an edge of the network, ordered from highest to lowest edge-weight; **c**: Bootstrapped difference test for node Strength centrality in the network of left-behind children. Black boxes indicate strengths that are significantly different from one another, and grey boxes indicate strengths that are not significantly different; **d**: Bootstrapped difference test for node Closeness centrality in the network of left-behind children. Black boxes indicate closeness that are significantly different from one another, and grey boxes indicate closeness that are not significantly different; **e**: Bootstrapped difference test for node Betweenness in the network of all left-behind children. Black boxes indicate betweenness that are significantly different from one another, and grey boxes indicate betweenness that are not significantly different; **f**: Bootstrapped difference tests between edge-weights in the network of left-behind children. Black boxes indicate edges that were significantly different from one another. Grey boxes indicate edges that were not significantly different.


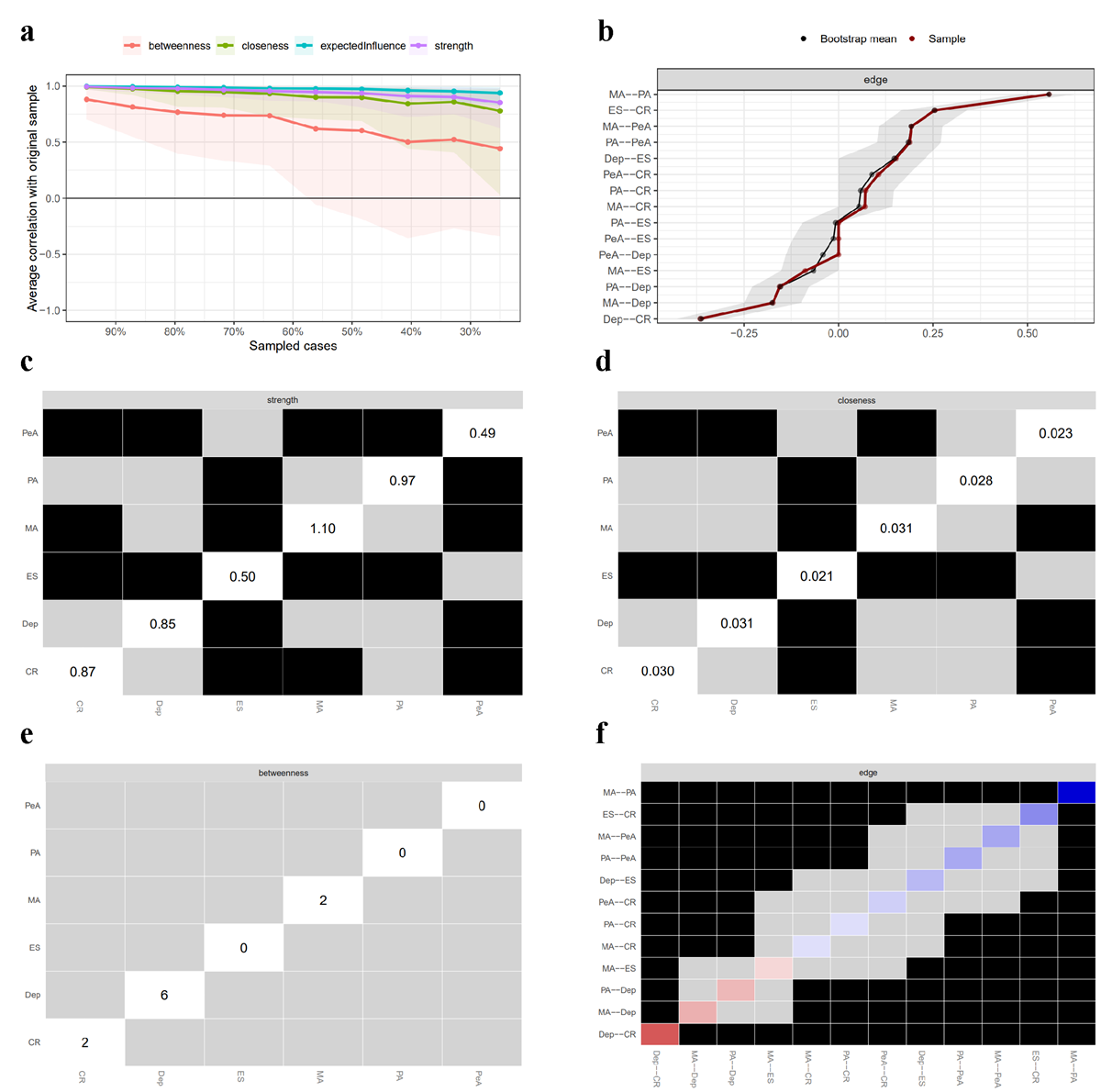


**Figure S3.** Stability and accuracy of the network for non-left-behind children. **a**: Different colored lines represent the average correlation between strength, closeness, betweenness, or EI within the complete sample and the subsample, respectively. The red area indicates the range from the 2.5th percentile to the 97.5th percentile; **b**: Bootstrapped confidence intervals of estimated edge-weights for non-left-behind children network. The red line indicates the values of edge-weight estimated in the whole sample, the grey area surrounding the red line represents the 95% quantile of the bootstrapped sampling distribution, and each horizontal line represents an edge of the network, ordered from highest to lowest edge-weight; **c**: Bootstrapped difference test for node Strength centrality in the network of non-left-behind children. Black boxes indicate strengths that are significantly different from one another, and grey boxes indicate strengths that are not significantly different; **d**: Bootstrapped difference test for node Closeness centrality in the network of non-left-behind children. Black boxes indicate closeness that are significantly different from one another, and grey boxes indicate closeness that are not significantly different; **e**: Bootstrapped difference test for node Betweenness in the network of non-left-behind children. Black boxes indicate betweenness that are significantly different from one another, and grey boxes indicate betweenness that are not significantly different; **f**: Bootstrapped difference tests between edge-weights in the network of non-left-behind children. Black boxes indicate edges that were significantly different from one another. Grey boxes indicate edges that were not significantly different.

*
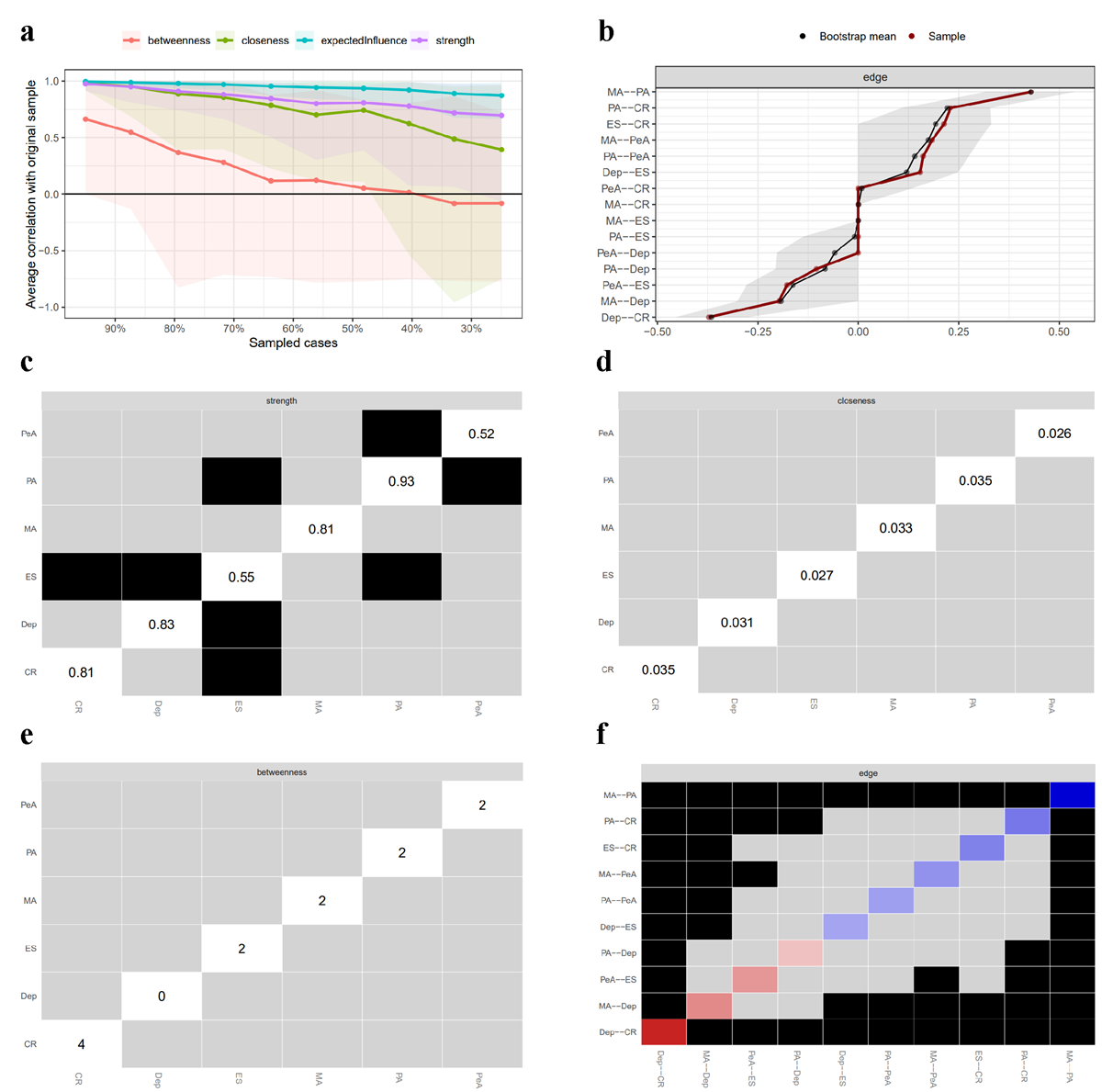
*

**Figure S4.** Stability and accuracy of the network for father absent group. **a**: Different colored lines represent the average correlation between strength, closeness, betweenness, or EI within the complete sample and the subsample, respectively. The red area indicates the range from the 2.5th percentile to the 97.5th percentile; **b**: Bootstrapped confidence intervals of estimated edge-weights for father absent group network. The red line indicates the values of edge-weight estimated in the whole sample, the grey area surrounding the red line represents the 95% quantile of the bootstrapped sampling distribution, and each horizontal line represents an edge of the network, ordered from highest to lowest edge-weight; **c**: Bootstrapped difference test for node Strength centrality in the network of father absent group. Black boxes indicate strengths that are significantly different from one another, and grey boxes indicate strengths that are not significantly different; **d**: Bootstrapped difference test for node Closeness centrality in the network of father absent group. Black boxes indicate closeness that are significantly different from one another, and grey boxes indicate closeness that are not significantly different; **e**: Bootstrapped difference test for node Betweenness in the network of father absent group. Black boxes indicate betweenness that are significantly different from one another, and grey boxes indicate betweenness that are not significantly different; **f**: Bootstrapped difference tests between edge-weights in the network of father absent group. Black boxes indicate edges that were significantly different from one another. Grey boxes indicate edges that were not significantly different.

*
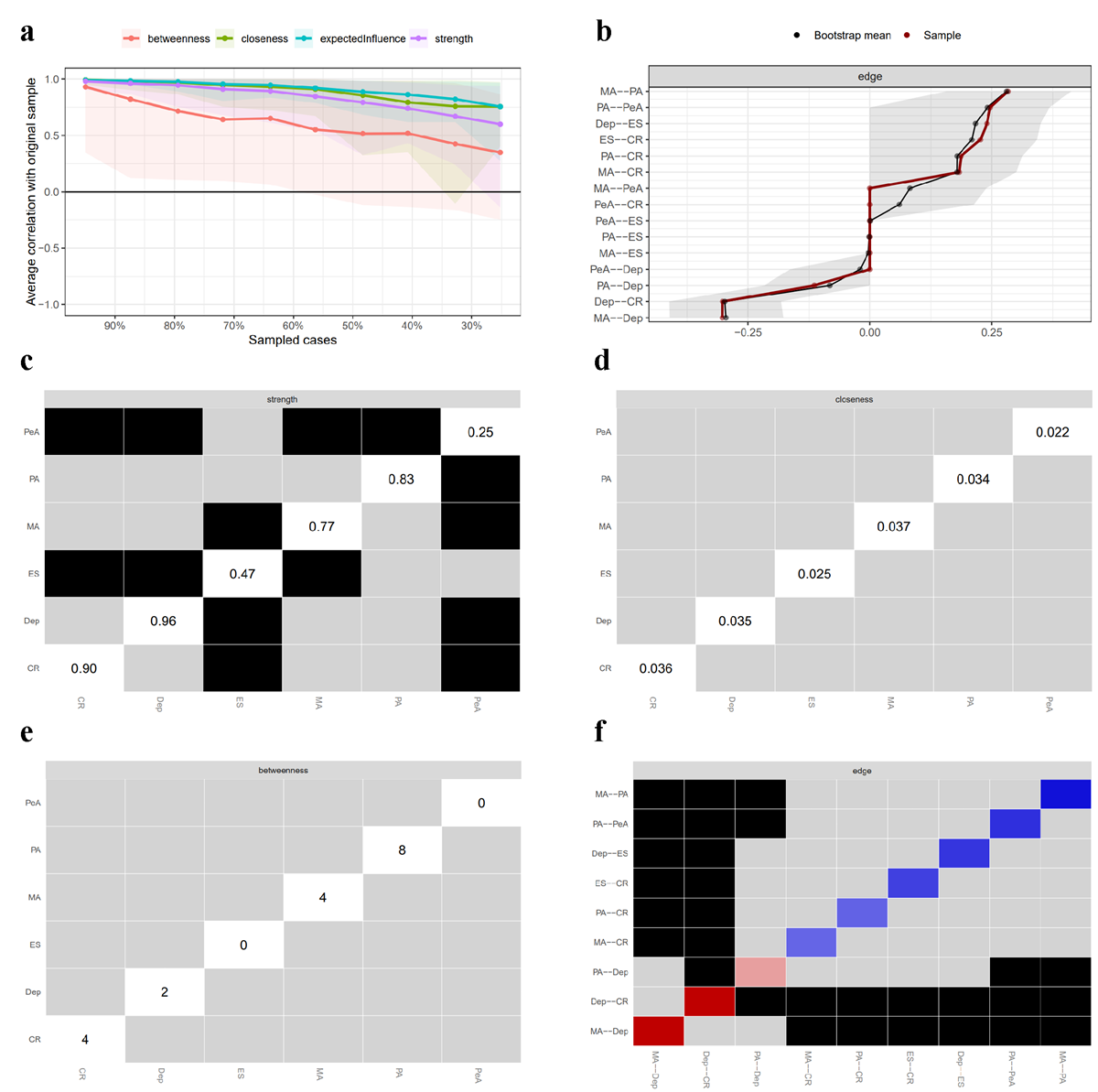
*

**Figure S5.** Stability and accuracy of the network for father & mother absent group. **a**: Different colored lines represent the average correlation between strength, closeness, betweenness, or EI within the complete sample and the subsample, respectively. The red area indicates the range from the 2.5th percentile to the 97.5th percentile; **b**: Bootstrapped confidence intervals of estimated edge-weights for father & mother absent group network. The red line indicates the values of edge-weight estimated in the whole sample, the grey area surrounding the red line represents the 95% quantile of the bootstrapped sampling distribution, and each horizontal line represents an edge of the network, ordered from highest to lowest edge-weight; **c**: Bootstrapped difference test for node Strength centrality in the network of father & mother absent group. Black boxes indicate strengths that are significantly different from one another, and grey boxes indicate strengths that are not significantly different; **d**: Bootstrapped difference test for node Closeness centrality in the network of father & mother absent group. Black boxes indicate closeness that are significantly different from one another, and grey boxes indicate closeness that are not significantly different; **e**: Bootstrapped difference test for node Betweenness in the network of father & mother absent group. Black boxes indicate betweenness that are significantly different from one another, and grey boxes indicate betweenness that are not significantly different; **f**: Bootstrapped difference tests between edge-weights in the network of father & mother absent group. Black boxes indicate edges that were significantly different from one another. Grey boxes indicate edges that were not significantly different.

## *5.5 Centrality of the network*

**Table S3**. Centrality of nodes in the left-behind children network (n = 573).

|  | **Strength** | **Closeness** | **Betweenness** | **EI** |
| --- | --- | --- | --- | --- |
| Maternal attachment | .79 | .04 | 2 | .27 |
| Paternal attachment | .82 | .03 | 2 | .65 |
| Peer attachment | .44 | .02 | 0 | .26 |
| Cognitive reappraisal | .81 | .03 | 2 | .03 |
| Expressive suppression | .42 | .02 | 0 | .42 |
| Depression | 1.01 | .04 | 2 | -.63 |

**Note:** EI, expected influence.

**Table S4**. Centrality of nodes in the non-left-behind children network (n = 552).

|  | **Strength** | **Closeness** | **Betweenness** | **EI** |
| --- | --- | --- | --- | --- |
| Maternal attachment | 1.08 | .03 | 1 | .56 |
| Paternal attachment | .97 | .03 | 0 | .66 |
| Peer attachment | .49 | .02 | 0 | .49 |
| Cognitive reappraisal | .87 | .03 | 1 | .14 |
| Expressive suppression | .50 | .02 | 0 | .32 |
| Depression | .85 | .03 | 3 | -.54 |

**Note:** EI, expected influence.

**Table S5**. Centrality of nodes in the father absent group network (n = 301).

|  | **Strength** | **Closeness** | **Betweenness** | **EI** |
| --- | --- | --- | --- | --- |
| Maternal attachment | .81 | .03 | 1 | .42 |
| Paternal attachment | .93 | .03 | 1 | .72 |
| Peer attachment | .52 | .03 | 1 | .17 |
| Cognitive reappraisal | .81 | .03 | 2 | .07 |
| Expressive suppression | .55 | .03 | 1 | .19 |
| Depression | .83 | .03 | 0 | -.52 |

**Note:** EI, expected influence.

**Table S6**. Centrality of nodes in the father & mother absent group network (n = 238).

|  | **Strength** | **Closeness** | **Betweenness** | **EI** |
| --- | --- | --- | --- | --- |
| Maternal attachment | .77 | .04 | 2 | .16 |
| Paternal attachment | .83 | .03 | 4 | .60 |
| Peer attachment | .25 | .02 | 0 | .25 |
| Cognitive reappraisal | .90 | .04 | 2 | .29 |
| Expressive suppression | .47 | .03 | 0 | .47 |
| Depression | .96 | .04 | 1 | -.48 |

**Note:** EI, expected influence.

## *5.6 Differences in edge-weights of the networks*

**Table S7.** Differences in edge-weights of the networks for NLBC (n = 552) and LBC (n = 573).

| **Edges** | **Edge - weights** | **Edge - weights** | ***p*** |
| --- | --- | --- | --- |
|  | **（NLBC）** | **（LBC）** |  |
| MA - PA | .58 | .37 | .01 |
| MA - PeA | .19 | .17 | 1 |
| MA - CR | .08 | .05 | 1 |
| MA - ES | -.10 | 0 | .56 |
| MA - Dep | -.17 | -.26 | 1 |
| PA - PeA | .19 | .18 | 1 |
| PA - CR | .07 | .20 | .56 |
| PA - ES | -.02 | -.07 | 1 |
| PA - Dep | -.15 | -.07 | 1 |
| PeA - CR | .12 | .06 | 1 |
| PeA - ES | -.06 | -.10 | 1 |
| PeA - Dep | -.05 | -.09 | 1 |
| CR - ES | .33 | .29 | 1 |
| CR - Dep | -.40 | -.41 | 1 |
| ES - Dep | .20 | .22 | 1 |

**Note**: The Network Comparison Test (NCT), a two-tailed permutation test, was conducted to examine the differences between the edge-weights of two networks. P^a^ indicated significant level of the edge-weight differences between Control group and Father Absent group. P-values was corrected using Holm-Bonferroni. NLBC, non-left-behind children; LBC, left-behind children; MA, maternal attachment; PA, paternal attachment; PeA, peer attachment; CR, cognitive reappraisal; ES, expressive suppression; Dep, Depression.

**Table S8.** Differences in edge-weights of the networks for NLBC group (n = 552), Father Absent group (n = 301) and Father & Mother Absent group (n = 238).

| **Edges** | **Edge - weights** | **Edge - weights** | **Edge - weights** | ***p*^a^** | ***p*^b^** | ***p*^c^** |
| --- | --- | --- | --- | --- | --- | --- |
|  | **（NLBC）** | **（Father Absent）** | **（Father & Mother Absent）** |  |  |  |
| MA - PA | .58 | .44 | .28 | .69 | .01 | 1 |
| MA - PeA | .19 | .19 | .11 | 1 | 1 | 1 |
| MA - ES | -.10 | 0 | 0 | 1 | 1 | 1 |
| MA - CR | .08 | 0 | .18 | 1 | 1 | .13 |
| MA - Dep | -.17 | -.20 | -.30 | 1 | 1 | 1 |
| PA - PeA | .19 | .16 | .24 | 1 | 1 | 1 |
| PA - ES | -.02 | -.05 | 0 | 1 | 1 | 1 |
| PA - CR | .07 | .24 | .18 | .25 | 1 | 1 |
| PA - Dep | -.15 | -.10 | -.12 | 1 | 1 | 1 |
| PeA - ES | -.06 | -.19 | 0 | 1 | 1 | .42 |
| PeA - CR | .12 | .04 | .1 | 1 | 1 | 1 |
| PeA - Dep | -.05 | -.09 | -.05 | 1 | 1 | 1 |
| CR - ES | .33 | .26 | .19 | 1 | 1 | 1 |
| CR - Dep | -.40 | -.39 | -.29 | 1 | 1 | 1 |
| ES - Dep | .20 | .18 | .22 | 1 | 1 | 1 |

**Note**: The Network Comparison Test (NCT), a two-tailed permutation test, was conducted to examine the differences between the edge-weights of two networks. P^a^ indicated significant level of the edge-weight differences between Control group and Father Absent group. P^b^ indicated significant level of the edge-weight differences between Control group and Father & Mother Absent. P^c^ indicated significant level of the edge-weight differences between Father Absent group and Father & Mother Absent group. All p-values were corrected using Holm-Bonferroni. MA, maternal attachment; PA, paternal attachment; PeA, peer attachment; CR, cognitive reappraisal; ES, expressive suppression; Dep, Depression.

## *5.7 Results all path coefficients in the chain mediation models*

**Table S9.** Examination of the mediating model of MA→CR and PA→CR.

| **Result variables** | **Predictors** | ***R^2^*** | ***F(df)*** | ***β*** | ***t*** | ***p*** |
| --- | --- | --- | --- | --- | --- | --- |
| **MA**→**CR** | | | | | | |
| MA | Left-behind experience | .005 | 5.30  (1, 1123) | -.14 | -2.30 | .022 |
| CR | Left-behind experience | .17 | 110.23  (2, 1122) | -.07 | -1.23 | .218 |
|  | MA |  |  | .40 | 14.68 | < .001 |
| Depression | Left-behind experience | .40 | 243.67  (3, 1121) | .13 | 2.72 | .007 |
|  | MA |  |  | -.38 | -14.73 | < .001 |
|  | CR |  |  | -.37 | -14.38 | < .001 |
| **PA**→**CR** | | | | | | |
| PA | Left-behind experience | .12 | 15.78  (1, 1123) | -.24 | -3.97 | < .001 |
| CR | Left-behind experience | .18 | 124.75  (2, 1122) | -.02 | -.41 | .681 |
|  | PA |  |  | .43 | 15.63 | < .001 |
| Depression | Left-behind experience | .36 | 213.14  (3, 1121) | .10 | 2.72 | .038 |
|  | PA |  |  | -.33 | -14.73 | < .001 |
|  | CR |  |  | -.38 | -14.38 | < .001 |

**Note:** MA, maternal attachment; PA, paternal attachment; CR, cognitive reappraisal.

**Table S10.** Examination of the mediating model of MA→ES and PA→ES.

| **Result variables** | **Predictors** | ***R^2^*** | ***F(df)*** | ***β*** | ***t*** | ***p*** |
| --- | --- | --- | --- | --- | --- | --- |
| **MA**→**ES** | | | | | | |
| MA | Left-behind experience | .005 | 5.30  (1, 1123) | -.14 | -2.30 | .022 |
|  | | | | | |  |
| ES | Left-behind experience | .02 | 12.71  (2, 1122) | .11 | 1.91 | .056 |
|  | MA |  |  | -.13 | -4.52 | < .001 |
|  | | | | | |  |
| Depression | Left-behind experience | .29 | 155.23  (3, 1121) | .14 | 2.77 | .006 |
|  | MA |  |  | -.51 | -19.99 | < .001 |
|  | ES |  |  | .10 | 4.08 | < .001 |
| **PA**→**ES** | | | | | | |
| PA | Left-behind experience | .01 | 15.78  (1, 1123) | -.24 | -3.97 | < .001 |
|  | | | | | |  |
| ES | Left-behind experience | .02 | 13.91  (2, 1122) | .10 | 1.65 | .099 |
|  | PA |  |  | -.14 | -4.78 | < .001 |
|  | | | | | |  |
| Depression | Left-behind experience | .26 | 128.93  (3, 1121) | .10 | 1.89 | .059 |
|  | PA |  |  | -.47 | -18.00 | < .001 |
|  | ES |  |  | .11 | 4.04 | < .001 |

**Note:** MA, maternal attachment; PA, paternal attachment; ES, expressive suppression.

# Reference

Borsboom, D., & Cramer, A. O. J. (2013). Network analysis: An integrative approach to the structure of psychopathology. *Annual Review of Clinical Psychology*, *9*(1), 91-121. <https://doi.org/10.1146/annurev-clinpsy-050212-185608>

Hu, H.-x., Liu, C., Zhang, J.-b., Wang, L.-l., Chu, M.-y., Li, S.-b.,…Chan, R. C. K. (2023). A transdiagnostic network analysis of motivation and pleasure, expressivity and social functioning. *Nature Mental Health*, *1*(8), 586-595. <https://doi.org/10.1038/s44220-023-00102-3>

van Borkulo, C., Boschloo, L., Borsboom, D., Penninx, B. W. J. H., Waldorp, L. J., & Schoevers, R. A. (2015). Association of symptom network structure with the course of depression. *Journal of the American Medical Association Psychiatry*, *72*(12), 1219-1226. <https://doi.org/10.1001/jamapsychiatry.2015.2079>

Zhou, J., Zhou, J., Feng, L., Feng, Y., Xiao, L., Chen, X.,…Wang, G. (2022). The associations between depressive symptoms, functional impairment, and quality of life, in patients with major depression: Undirected and Bayesian network analyses. *Psychological Medicine*, *53*(14), 6446-6458. <https://doi.org/10.1017/s0033291722003385>
